# Imbalance in Carbon and Nitrogen Metabolism in *Comamonas testosteroni* R2 Is Caused by Negative Feedback and Rescued by L-arginine

**DOI:** 10.1264/jsme2.ME21050

**Published:** 2021-10-13

**Authors:** Abd Rahman Jabir Mohd Din, Kenshi Suzuki, Masahiro Honjo, Koki Amano, Tomoka Nishimura, Ryota Moriuchi, Hideo Dohra, Hidehiro Ishizawa, Motohiko Kimura, Yosuke Tashiro, Hiroyuki Futamata

**Affiliations:** 1 Graduate School of Science and Technology, Shizuoka University, Hamamatsu, Hamamatsu 432–8011, Japan; 2 Innovation Centre in Agritechnology for Advanced Bioprocess, UTM Pagoh Research Center, 84600 Muar, Johor, Malaysia; 3 Microbial Ecotechnology (Social Cooperation Laboratory), Department of Biotechnology, Graduate School of Agricultural and Life Sciences, The University of Tokyo, Bunkyo-ku, Tokyo, 113–8654, Japan; 4 Department of Applied Chemistry and Biochemical Engineering, Graduate School of Engineering, Shizuoka University, Hamamatsu, 432–8011, Japan; 5 Research Institution of Green Science and Technology, Shizuoka University, Shizuoka 422–8529, Japan

**Keywords:** metabolism, negative feedback, ammonium, L-arginine, *Comamonas*

## Abstract

The collapse of *Comamonas testosteroni* R2 under chemostat conditions and the aerobic growth of strain R2 under batch conditions with phenol as the sole carbon source were investigated using physiological and transcriptomic techniques. Phenol-/catechol-degrading activities under chemostat conditions gradually decreased, suggesting that metabolites produced from strain R2 accumulated in the culture, which caused negative feedback. The competitive inhibition of phenol hydroxylase and catechol dioxygenase was observed in a crude extract of the supernatant collected from the collapsed culture. Transcriptomic analyses showed that genes related to nitrogen transport were up-regulated; the ammonium transporter *amtB* was up-regulated approximately 190-fold in the collapsed status, suggesting an increase in the concentration of ammonium in cells. The transcriptional levels of most of the genes related to gluconeogenesis, glycolysis, the pentose phosphate pathway, and the TCA and urea cycles decreased by ~0.7-fold in the stable status, whereas the activities of glutamate synthase and glutamine synthetase increased by ~2-fold. These results suggest that ammonium was assimilated into glutamate and glutamine via 2-oxoglutarate under the limited supply of carbon skeletons, whereas the synthesis of other amino acids and nucleotides was repressed by 0.6-fold. Furthermore, negative feedback appeared to cause an imbalance between carbon and nitrogen metabolism, resulting in collapse. The effects of amino acids on negative feedback were investigated. L-arginine allowed strain R2 to grow normally, even under growth-inhibiting conditions, suggesting that the imbalance was corrected by the stimulation of the urea cycle, resulting in the rescue of strain R2.

It has been challenging to understand the principles by which microbial communities are formed (Fernandez *et al.*, 1999, 2000; Haruta *et al.*, 2013; El-Chakhtoura *et al.*, 2015). Microbial diversity, the functional stability of whole systems, and the coexistence of different microbes (Aziz *et al.*, 2015; Azwani *et al.*, 2021) are important features of microbial ecosystems and are relevant to the mechanisms by which they are formed. The functional stability of microbial communities is important for agricultural production, wastewater treatment, bioremediation, human health, and ecosystems, and is based on metabolic processes in response to changing environmental conditions.

Real microbial ecosystems are too complex to analyze because of intertwined relationships based on the functional diversity of the microbial world. Therefore, more simple and controllable systems, known as synthetic bacterial communities (SBCs), are needed. SBCs have increasing become the focus of research in recent years due to the reduced complexity of natural ecosystems and increased controllability (Haruta *et al.*, 2002; Kato *et al.*, 2005; Narisawa *et al.*, 2008; De Roy *et al.*, 2014; He *et al.*, 2014; Mee *et al.*, 2014; Aziz *et al.*, 2015; Friedman *et al.*, 2017; Haruta and Yamamoto, 2018). In our previous study, the SBC constructed with phenol-degrading bacteria, *Pseudomonas* sp. strain LAB-08, *Cupriavidus* sp. strain P-10, and *Comamonas testosteroni* strain R2, showed functional stability with coexistence under chemostat conditions for more 800 days, and this was predicted to be dependent on a metabolic networking system (Azwani *et al.*, 2021).

Metabolic networking systems are attracting the attention of researchers interested in the mechanisms by which microbial ecosystems are formed (Freilich *et al.*, 2011; Morris *et al.*, 2013; Cao *et al.*, 2018; Hsu *et al.*, 2019). Interspecies interactions are considered to be relevant to the formation of metabolic networks. The supernatants of microbial cultures may affect the metabolic processes of other microbes (Tanaka *et al.*, 2005; Tashiro *et al.*, 2013; Inaba *et al.*, 2015), in which microbial metabolites play major roles in positive (Christensen *et al.*, 2002) and negative interactions (Kim and Copley, 2012). In our efforts to clarify the coexisting mechanisms of three strains, LAB-08, P-10, and R2, we found a unique phenomenon. A pure culture of strain R2 suddenly collapsed under chemostat conditions supplied with phenol as the sole carbon and energy source, even though strain R2 has a complete set of genes relevant to phenol-utilizing metabolism for the conversion of phenol to acetyl-CoA (Powlowski and Shingler, 1994; Azwani *et al.*, 2017) and grew and completely degraded phenol under aerobic batch conditions with phenol as the sole carbon and energy source (Watanabe *et al.*, 1996; Futamata *et al.*, 2001a). These findings suggest that the three strains coexist through mutualistic interactions. Therefore, analyses of the collapsing process occurring in strain R2 will provide insights into coexisting mechanisms, how the metabolic network is formed, and how to manage microbes.

The aims of the present study were to analyze collapsing processes using physiological and transcriptomic techniques and to develop a method that rescues strain R2 from collapse. Physiological analyses indicated that collapse was induced by feedback growth inhibition, which had already been initiated under stable conditions. The transcriptomic analysis showed that the activities of primary metabolism decreased by approximately 60 to 70% of stable conditions. An imbalance between carbon and nitrogen metabolism appeared to occur due to a decrease in the supply of carbon skeletons and an increase in ammonium influx into cells. We herein also discuss how strain R2 responded to negative feedback, the mechanisms underlying the imbalance in carbon and nitrogen metabolism in cells, and how strain R2 was rescued from collapse.

## Materials and Methods

### Bacterium and culture conditions

The phenol-degrading bacterium *C. testosteroni* strain R2 was used in the present study. Strain R2 was isolated from activated sludge in wastewater treatment at an oil refinery plant (Watanabe *et al.*, 1996). Strain R2 was precultured at 25°C in BSM medium supplemented with phenol at 2.0‍ ‍mM (BSM2.0phe medium) under aerobic and batch conditions (Futamata *et al.*, 2001a). The medium contained the following (L^–1^): 12.5‍ ‍g K_2_HPO_4_, 3.8‍ ‍g KH_2_PO_4_, 1.0‍ ‍g (NH_4_)_2_SO_4_, 0.1‍ ‍g MgSO_4_·7H_2_O, and 5‍ ‍mL of trace-element solution (pH 7.2). The trace-element solution contained the following (L^–1^): 0.232‍ ‍g H_3_BO_3_, 0.174‍ ‍g ZnSO_4_·7H_2_O, 0.116‍ ‍g Fe(NH_4_)_2_(SO_4_)_2_·6H_2_O, 0.096‍ ‍g CoSO_4_·7H_2_O, 0.022‍ ‍g (NH_4_)_6_Mo_7_O_24_·4H_2_O, 8‍ ‍mg CuSO_4_·5H_2_O, and 8‍ ‍mg MnSO_4_·4H_2_O. Cultures were harvested at the mid-exponential growth phase and then transferred to 1.5 L of BSM medium containing 0.2‍ ‍mM of phenol in a chemostat reactor (capacity of 2 L). The initial cell density of strain R2 was adjusted to approximately 1.0×10^5^‍ ‍cells‍ ‍mL^–1^ by measuring optical density at 600‍ ‍nm (OD_600‍ ‍nm_). OD_600‍ ‍nm_ of 0.1 corresponded to 5.0×10^8^‍ ‍cells‍ ‍mL^–1^. After the added phenol had almost been completely degraded (the start-up phase), the chemostat culture was continuously supplied with BSM medium containing phenol (1,500‍ ‍mg L^–1^) at a flow rate of 6.25‍ ‍mL h^–1^, which corresponded to a dilution rate (*D*) of 0.1 day^–1^ (6.25‍ ‍mL h^–1^×24 h/1,500‍ ‍mL). The culture volume was maintained at 1.5 L. The hydraulic residence time (HRT), calculated as 1/*D*, was 10 days. The culture was stirred at 150‍ ‍rpm, and temperature and pH were maintained at 25°C and 7.0, respectively. Air was filtered through membrane filters with a pore size of 0.2‍ ‍μm (Merck Millipore) and supplied to the culture at 1.5‍ ‍L‍ ‍min^–1^. Phenol in the culture was checked using a colorimetric assay with the Phenol Test Wako kit (FUJIFILM Wako Pure Chemical) (Futamata *et al.*, 2001b), which has a detection limit of approximately 1.0‍ ‍μM. One milliliter of the cell suspension was taken from the chemostat culture and centrifuged at 5,800×*g* at 4°C for 5‍ ‍min. The pellet was resuspended in 1‍ ‍mL of 0.85% NaCI solution (w/v). The sample was incubated at room temperature for 15‍ ‍min in the dark to stain cells with the LIVE/DEAD^®^ BacLight^TM^ bacterial viability kit L7007 (Molecular Probes) according to the manufacturer’s instructions. Samples were analyzed using the fluorescence microscope Olympus IX73 (Olympus). Stained cells were counted using ImageJ software. Cell viability was calculated as the percentage of live cells among the total cell number.

### Relative growth activity

The effects of feedback growth inhibition on strain R2 were investigated using a supernatant collected from a pure chemostat culture and evaluated as specific growth activity. Growth curves were recorded to estimate the physiological changes that occurred after the addition of the supernatant. Strain R2 was incubated in BSM medium under the conditions of the chemostat culture supplemented with phenol as the sole carbon source. The culture was sampled and centrifuged at 5,800×*g* at 4°C with adequate intervals. The supernatant was sterilized by filtration through a Steriflip-GP Filter (pore size of 0.22‍ ‍μm, Millipore). Strain R2 was precultured in BSM medium supplemented with 2.0‍ ‍mM phenol (BSM2.0phe) and 0.3‍ ‍mL of filter-sterilized supernatant were transferred into 2.7‍ ‍mL of fresh BSM2.0phe medium. The initial amount of cells was adjusted to OD_600‍ ‍nm_ of 0.01. As the control condition, 0.3‍ ‍mL of BSM medium without phenol was added instead of the supernatant. The growth curve was automatically measured using a Bio-photorecorder (TVS062CA, ADVANTEC). Growth parameters, including the lag time (h), growth rate constant (*μ* [h^–1^]), and amount of growth in the stationary phase (*OD*_max_), were calculated using the growth curve. We herein defined specific growth activity as surviving activity maintaining cell density at more than 1.0×10^9^‍ ‍cells‍ ‍mL^–1^ in a chemostat culture under the condition of *D*. Therefore, 1 unit (U) of specific growth activity was calculated using the following equation: 1 U=0.105 (h^–1^)×10^9^ (cells mL^–1^) under HRT of 10 days. As described above, the cell density of strain R2 was 5.0×10^8^‍ ‍cells‍ ‍mL^–1^ at an OD_600‍ ‍nm_ of 0.1. *OD*_max_ was then converted to cell density. The unit of specific growth activity was calculated according to the following equation: U=(μ×*cell density from ODmax*)/(1 U×*lag time*) (Aziz *et al.*, 2015). The proportion of U in the presence of the supernatant (U_sup_) to U in the control condition (U_cont_) was calculated as relative growth activity (%).

### Real-time quantitative PCR (qPCR)

The population density of strain R2 was monitored using real-time qPCR targeting the gene encoding the large subunit of phenol hydroxylase (PH). Specific sets of primers were designed by the alignment of genes encoding the large subunit of PH in strain R2 (Azwani *et al.*, 2021). A specific PCR product amplified with a specific primer set was used as the standard DNA fragment in the qPCR analysis. To monitor strain R2, the qPCR profile consisted of preheating at 95°C for 10‍ ‍min, followed by 40 cycles of denaturation at 95°C for 10‍ ‍s, annealing at 63°C for 5‍ ‍s, and extension at 72°C for 15 s. The fluorescence signal was detected at 72°C in each cycle, and a melting curve was obtained by heating the product to 95°C and cooling to 40°C. The reaction was performed using a LightCycler FastStart DNA Master SYBR Green I kit (Roche Molecular Biochemicals) and a LightCycler system (Roche Diagnostics) according to the manufacturer’s instructions. The copy number of each amplicon was calculated using LightCycler software version 3.52. The copy number of the amplicon was equal to the cell number because only one copy of the PH gene was present in all strains (Azwani *et al.*, 2017).

### Kinetic analysis

A kinetic analysis was conducted to investigate the effects of metabolites on phenol- and catechol-degrading activities. Strain R2 was grown in a chemostat reactor with BSM medium and phenol as the sole carbon and energy source (R2-chemostat), and the kinetic properties of strain R2 for phenol and catechol degradation were investigated using the culture on days 18, 25, 30, and 35 according to a previously described method (Futamata *et al.*, 2001a; Haruta *et al.*, 2013). When the accumulation of phenol and a decrease in OD_600‍ ‍nm_ were observed in the R2-chemostat, we considered the system to be collapsed. In total, 1.5 L of the culture was collected from the collapsed R2-chemostat and centrifuged at 5,800×*g* at 4°C. The supernatant was treated with the same volume of hexane, ethyl acetate, butanol, and dH_2_O. Organic solvent fractions were concentrated to approximately 60‍ ‍mL using a rotary evaporator (Buchi Rotavapor R3) under vacuum conditions (Buchi V-700) at 40°C. The H_2_O fraction was mixed with methanol at a ratio of 1:3 and kept overnight at 4°C. The solution was then centrifuged at 5,800×*g* at 4°C and filtered (0.2‍ ‍μm PTFE Membrane, OmnipureTM, Merck Millipore) to remove precipitates. The H_2_O fraction was concentrated to approximately 60‍ ‍mL by evaporating methanol using the rotary evaporator under vacuum conditions at 40°C. When the effects of the H_2_O fraction on kinetic parameters were investigated, the culture of strain R2 and the H_2_O fraction were mixed at a ratio of 9:1 for 30‍ ‍min. Phenol- and catechol-oxygenating activities (the phenol and catechol consumption rates) were measured at various phenol and catechol concentrations, respectively, using an oxygen electrode (DO METER TD-51, Toko Chemical Lab.) after respiratory oxygen consumption had been suppressed by the addition of potassium cyanide (Watanabe *et al.*, 1996). Kinetic parameters were calculated using the initial phenol-oxygenating velocities at more than 10 different substrate concentrations. Data were fit to the Michaelis-Menten or Haldane equation (Folsom *et al.*, 1990; Watanabe *et al.*, 1998; Futamata *et al.*, 2005) using JMP statistical visualization software (SAS Institute). The apparent kinetic constants, *K*_S_ (affinity constant) and *V*_max_ (theoretical maximum activity) were assessed using the non-linear regression method as previously described (Azwani *et al.*, 2021). As reported by Folsom *et al.* (1990), the term *K*_S_ was employed instead of *K*_m_ because activity was measured using intact cells rather than purified enzymes.

### Transcriptomic analysis

A transcriptomic analysis was conducted to analyze contributing factors to the change from a stable to collapsed status in strain R2. Strain R2 was incubated in new chemostat cultures, reactors I and II, until collapse and cells were collected on days 17 (sample I-1), 22 (I-2), 30 (I-3), 31 (I-4), and 32 (I-5) from reactor I and on days 15 (sample II-1), 20 (II-2), 23 (II-3), 25 (II-4), and 28 (II-5) from reactor II (Supplementary Fig. S1). In the present study, samples I-1 and II-1 were defined as a stable status under which phenol did not accumulate and the population density was maintained, while samples I-5 and II-5 were defined as a collapsed status under which phenol accumulated. The library preparation and RNA sequencing of strain R2 were performed by Macrogen. The total RNA of strain R2 cultivated under chemostat conditions was extracted using the SV Total RNA Isolation System (Promega). The removal of ribosomal RNA molecules from total RNA was performed using the NEBNext rRNA Depletion Kit (Bacteria) (New England Biolabs) and strand-specific RNA sequencing libraries were prepared using TruSeq Stranded Total RNA Library Prep Gold (Illumina). Libraries were sequenced on NovaSeq 6000 (Illumina) to generate 2×101-bp paired-end sequence reads. Raw reads were cleaned using Trimmomatic ver. 0.36 by trimming adapter sequences, the base at the 3′-end, low-quality ends (quality score, <15), and dropping out reads of less than 75 bp (Bolger *et al.*, 2014). The resulting high-quality reads were aligned to the genome sequence of strain R2 (GenBank accession number BDQJ00000000.1) using HISAT2 ver. 2.1.0 with options --dta and --no-spliced-alignment (Kim *et al.*, 2019). Read counts were calculated from BAM files using featureCounts ver. 2.0.0 (Liao *et al.*, 2014) and transcripts per million (TPM) values were calculated to normalize gene lengths and total read counts. The differentially expressed genes (DEGs) of strain R2 in the stable and collapsed statuses (I-1 vs I-5, and II-1 vs II-5) were analyzed using edgeR package ver. 3.16.4 (Robinson *et al.*, 2010). Read counts for genes were filtered by removing genes with low expression levels with a count per million (CPM) value of less than 2, and were normalized with scaling factors calculated for the library sizes using the Trimmed Mean of M-values (TMM) method (Robinson and Oshlack, 2010; Robinson *et al.*, 2010). DEGs were defined by a log_2_ fold-change (log_2_FC) ≥2 (up-regulated) or ≤–2 (down-regulated) and a false discovery rate (FDR) <0.05. Raw reads for RNA-seq analyzed in the present study have been deposited in the DDBJ Sequence Read Archive (DRA) under the accession numbers DRR309243 to DRR309247 and DRR309248 to DRR209252 for samples I-1 to II-5, respectively (Supplementary Table S1).

### Growth of strain R2 in the presence of amino acids

We investigated whether the growth inhibition of strain R2 was rescued by the following amino acids: L-arginine, L-ornithine, L-citrulline, L-glutamate, and L-glutamine, because these amino acids are directly and indirectly related to the urea cycle. L-arginine, L-ornithine, and L-citrulline consisting of the urea cycle, and L-glutamate and L-glutamine are precursors for L-citrulline. Strain R2 cells precultured in BSM2.0phe liquid culture and 0.4‍ ‍mL of filter-sterilized supernatant collected from collapsed chemostat cultures were transferred into 3.6‍ ‍mL of fresh BSM2.0phe medium. The initial amount of cells was adjusted to 0.01 at OD_600‍ ‍nm_. A stock solution of amino acids (200‍ ‍mM) was prepared in BSM medium and the filter-sterilized stock solution was added at a final concentration of 10‍ ‍mM. As the control condition, 0.4‍ ‍mL of BSM medium without phenol was added instead of the supernatant and amino acid solution. The growth curve was automatically measured using a Bio-photorecorder (TVS062CA, ADVANTEC). Growth parameters, including the lag time (h), growth rate constant (*μ* [h^–1^]), and the amount of growth in the stationary phase (*OD*_max_), were calculated using the growth curve. As described above in the section on relative growth activity, the unit of specific growth activity (U) was calculated in the presence of phenol and an amino acid as a control (U_amino acid_) or in the presence of phenol, the amino acid, and the supernatant (U_amino acid+supernatant_). The negative growth effect (%) was calculated using the following equation:

(1–*U_amino acid_*⁄*U_amino acid+supernatant_*)×100.

### Scanning electron microscopy (SEM) observations

SEM was used to observe the morphology of *C. testosteroni* strain R2 on days 18 and 38 in the chemostat culture. Samples were fixed with a mixed solution of 25% (v/v) glutaraldehyde and 5% (v/v) formaldehyde for 2 h in potassium phosphate buffer (0.2‍ ‍M, pH 7.0) and dehydrated with ethanol with serially increasing concentrations (30, 50, 75, 95, and 99.5% of ethanol for 15‍ ‍min each) and then with 100% of butyl alcohol. The sample was mounted on an aluminium stub with double-sided carbon type, and sputter-coated with gold under argon at a thickness of 50‍ ‍Å in the Quick Auto Coater (SC-701AT, Sanyu Denshi) for 20‍ ‍s. Following coating, samples were imaged at different magnifications with a field emission scanning electron microscope (Model JSM-6335F, JEOL) at an acceleration voltage of 5 kV and working distance of 5‍ ‍mm.

### Chemical analysis

Phenol and catechol concentrations were monitored using high-pressure liquid chromatography (Waters Japan) equipped with a column (YMC-Triart C18 [150×2‍ ‍mm], YMC) and UV detector. Liquid samples collected from the chemostat culture were centrifuged and filtered (Millipore LG [pore size of 0.2‍ ‍μm, diameter of 13‍ ‍mm], Millipore). Liquid samples were eluted using 50% acetonitrile solution with 20‍ ‍mM ammonium acetate delivered at 0.1‍ ‍mL min^–1^, and elutes were monitored at 210‍ ‍nm. Phenol and catechol were identified according to their retention times of 7.2 and 5.2‍ ‍min, respectively. Concentrations were assessed by comparing the peak area with that of the cognate standard sample.

### Statistical analysis

DEGs were identified by the likelihood-ratio test implemented in the edgeR package. Other data were analyzed using the Student’s *t*-test. *P*=0.05 was considered to be significant.

## Results

### Collapse of strain R2 growth under chemostat conditions

**Fig. 1.**
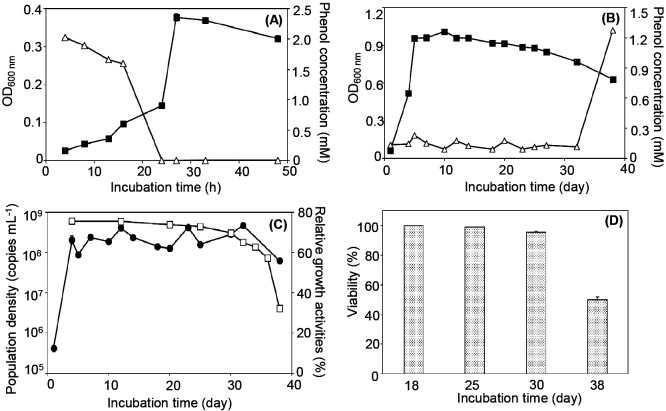
Growth of strain R2 under batch and chemostat conditions. (A) Growth of strain R2 under batch conditions. Open triangle: phenol concentration; Closed square: cell amount as the OD_600‍ ‍nm_ value. (B) Growth of strain R2 under chemostat conditions. Open triangle: phenol concentration; closed square: cell amount as the OD_600‍ ‍nm_ value. (C) Growth of strain R2 under chemostat conditions, which was the same reactor as that in (B). Open square: relative growth activity; closed circle: population density measured by qPCR. (D) Cell viability on days 18, 25, 30, and 38 in the chemostat culture.

Strain R2 grew aerobically in the presence of phenol as the sole carbon and energy source and completely utilized phenol in batch cultures (Fig. 1A). Catechol, a metabolite produced by PH, was not detected during the experiment (data not shown). In chemostat cultures, the growth amount of strain R2 reached 0.96±0.010 at OD_600‍ ‍nm_ from days 4 to 14 and then gradually decreased (Fig. 1B). The concentration of phenol was maintained at 0.13±0.010‍ ‍mM until day 32 and increased to 1.3‍ ‍mM±0.010 on day 38, namely, the growth of strain R2 collapsed (Fig. 1B). Relative growth activity was stable at 74±0.64% until day 25, after which it rapidly decreased to 32±0.080% on day 38 in the collapsed status (Fig. 1C). The viability of strain R2 cells decreased from 100±0.10% on day 18 to 50±1.8% on day 38 (Fig. 1D). SEM observations showed that the morphology of strain R2 cells changed in the collapsed status (Supplementary Fig. S2).

### Effects of the chemostat supernatant on kinetic properties

**Fig. 2.**
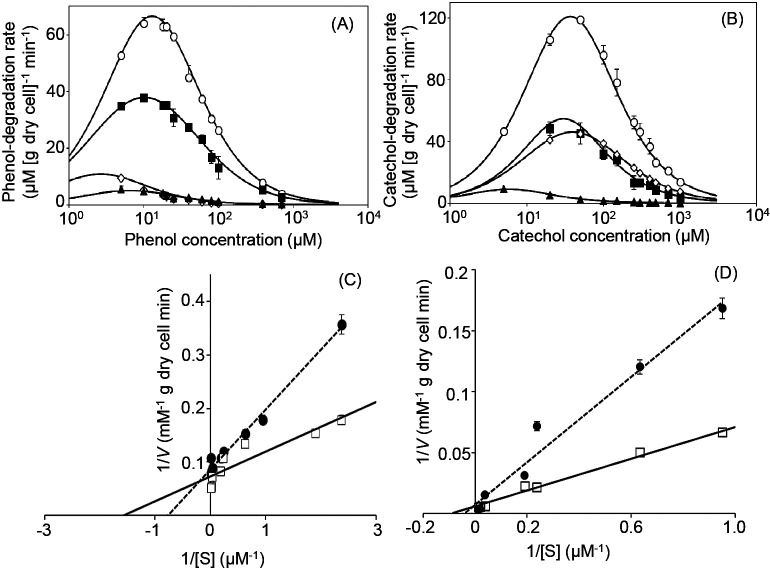
Effects of metabolites produced from strain R2 on kinetic properties for phenol and catechol degradation. (A and B): Kinetic parameters for phenol and catechol degradation by strain R2 were investigated using the R2-chemostat culture; (A): phenol; (B): catechol. The chemostat culture on days 18 (open circle), 25 (closed square), 30 (open diamond), and 35 (closed triangle) was used. (C and D): Lineweaver-Burk plot; (C): phenol; (D): catechol. Specific phenol- and catechol-degrading activities with only phenol as the control (open square), with phenol and the H_2_O fraction extracted from the collapsed supernatant (closed circle) being shown.

The kinetic properties of phenol and catechol degradation by strain R2 were investigated using R2-chemostat cultures on days 18, 25, 30, and 35. *V*_max_ for phenol and catechol were 65±6.4 and 130±13‍ ‍mM [g dry cell]^–1^, respectively, on day 18, and gradually decreased to 9.4±2.5 and 9.1±0.18‍ ‍mM [g dry cell]^–1^, respectively, on day 38 (Fig. 2A and B).

We hypothesized that certain metabolites accumulating in the chemostat culture of strain R2 may be responsible for the collapse of its growth. To clarify this, we investigated the effects of supernatant (SN) collected from chemostat cultures of strain R2 on kinetic parameters for phenol and catechol degradation. As a preliminary test, we fractionated the supernatant using H_2_O and organic solvents (*i.e.*, hexane, ethyl acetate and butanol) and found that the H_2_O fraction exerted the strongest negative effects on the relative growth activity of strain R2 (Supplementary Fig. S3). The H_2_O fraction of supernatant was used to establish whether the activities of PH and catechol dioxygenase were inhibited by metabolites produced from strain R2. *V*_max_ values were not markedly affected, whereas *K*_S_ values for PH and catechol dioxygenase increased from 0.70±0.050 to 1.3±0.10‍ ‍μM for phenol and from 14±1.4 to 36±0.70‍ ‍μM for catechol in the presence of the H_2_O fraction (Fig. 2C and D).

### Change in metabolism from the stable to collapsed status

The results of physiological analyses indicated that the growth collapse of strain R2 under chemostat conditions was triggered by metabolic changes. To elucidate the metabolic shift from the stable to collapsed status, we reproduced two chemostat cultures (namely, reactors I and II) of strain R2 under the same conditions and performed comparative transcriptomics. The population densities and phenol concentrations of strain R2 in chemostat reactors I and II slightly differed, but showed similar changes (Supplementary Fig. S1). We sequenced 10 samples, comprising 5 samples from each reactor in the stable (I-1 and II-1) and collapsed statuses (I-5 and II-5), and successfully retrieved *ca.* 41–54 million quality-filtered transcript reads per sample (Supplementary Table S1). We observed differences between the transcriptomic data obtained from reactors I and II (Supplementary Table S2, S3, S4, and S5). We hereafter explained potential metabolic changes in strain R2 based on the transcriptomic datasets obtained from reactors I and II. The top 20 up- and down-regulated genes in reactors I and II are shown in Table S5. Approximately 50% of the up-regulated genes were related to nitrogen metabolism in reactors I and II (Supplementary Table S5-1 and S5-2), *e.g.*, the expression of the ammonium transporter Amt family (CTR2_4688) and nitrate/nitrite transport system (CTR2_4163) was ~200-fold higher in the collapsed status (I-5 and II-5) than in the stable status (I-1 and II-1). On the other hand, common functional features were not observed among the down-regulated genes in reactors I and II (Supplementary Table S5-3 and S5-4). A ClueGo analysis (Bindea *et al.*, 2009, 2013) using all up- and down-regulated genes showed similar results, *i.e.*, nitrogen cycle metabolic processes were significantly up-regulated (Supplementary Fig. S4).

Relative transcriptional levels from the stable to collapsed status slightly decreased from 0.5- to 0.7-fold in the main metabolic pathways; glycolysis, gluconeogenesis, the pentose phosphate pathway, 5-phosphate-α-D-ribose 1-diphosphate (PRPP) synthesis (Supplementary Fig. S5 and Supplementary Table S2), and nucleotide synthesis (Supplementary Fig. S6 and Table S3). The relative transcriptional levels of genes encoding enzymes in the pentose phosphate pathway decreased to 0.67±0.20-fold in the collapsed status, whereas those of genes encoding PRPP decreased to 0.44-fold and 0.47-fold in the collapsed status in reactors I and II, respectively (Supplementary Fig. S5C).

**Fig. 3.**
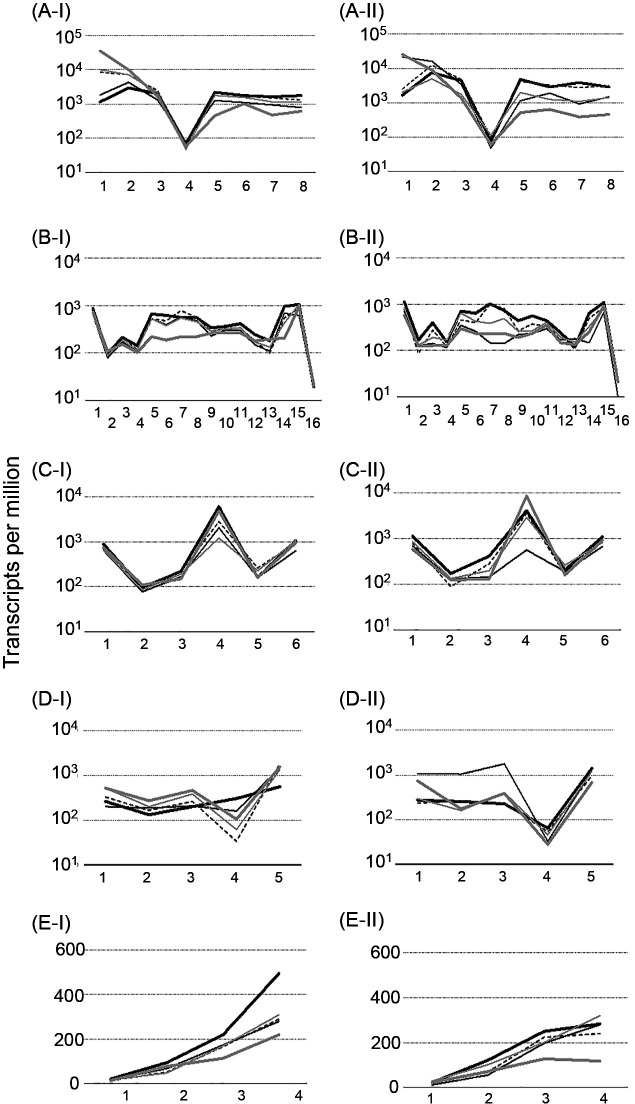
Relative transcriptional levels of genes encoding enzymes in phenol/catechol degradation, the TCA cycle, glyoxylate shunt, and urea cycle in reactors I and II. (A-I) and (A-II): Phenol/catechol degradation in reactors I and II, respectively; 1: from phenol to catechol; 2: from catechol to 2-hydroxymuconic semialdehyde; 3: from 2-hydroxymuconic semialdehyde to 2-hydroxymuconate; 4: from 2-hydroxymuconate to gamma-oxalocrotonate; 5: from gamma-oxalocrotonate to 2-oxopent-4-enoate; 6: from 2-oxopent-4-enoate to 4-hydroxy-2-oxopentanoate; 7: from 4-hydroxy-2-oxopentanoate to acetaldehyde/pyruvate; 8: from acetaldehyde/pyruvate to acetyl-CoA. (B-I) and (B-II): the TCA cycle in reactors I and II, respectively; 1: from acetyl-CoA and oxaloacetate to citrate; 2 and 3: from citrate to isocitrate; 4: from isocitrate to 2-oxoglutarate; 5: from 2-oxoglutarate to s-succinyl dihydrolipoylysine; 6: from s-succinyl dihydrolipoylysine to succinyl-CoA; 7 and 8: from succinyl-CoA to succinate; 9, 10, 11, and 12: from succinate to fumarate; 13 and 14: from fumarate to malate; 15 and 16: from malate to oxaloacetate. (C-I) and (C-II): the glyoxylate shunt in reactors I and II, respectively; 1: from acetyl-CoA and oxaloacetate to citrate; 2 and 3: from citrate to isocitrate; 4: from isocitrate to glyoxylate; 5: from glyoxylate to malate; 6: from malate to oxaloacetate. (D-I) and (D-II): GS-GOGAT and GDH synthesis; 1: from L-glutamate to L-glutamine by glutamine synthetase [EC:6.3.1.2]; 2: from L-glutamine to L-glutamate; 3: from L-glutamine to L-glutamate; 4: from 2-OG and NH_3_ to L-glutamate; 5: from L-glutamate to 2-OG and NH_3_. Bold black line: stage I-1 or stage II-1; black line: stage I-2 or stage II-2; broken line: stage I-3 or stage II-3; gray line: stage I-4 or stage II-4; and bold gray line: stage I-5 or II-5. (E-I) and (E-II): the urea cycle in reactors I and II, respectively; 1: from L-argininosuccinate to L-arginine and fumarate; 2: L-arginine to L-ornithine and urea; 3: from L-ornithine to L-citrulline; 4: from L-citrulline and L-aspartate to L-argininosuccinate. The genes, locus tag numbers, enzymes, ko numbers, and transcripts per million values of genes are listed in Supplementary Table S4.

**Fig. 4.**
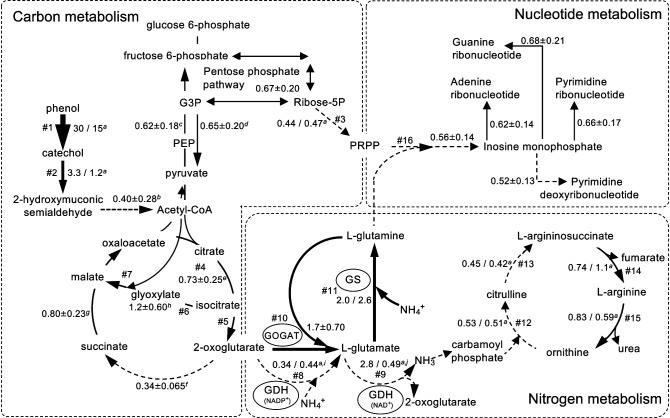
Schematic diagram of metabolism in strain R2 in the collapsed status. All values are shown as a percentage of the relative transcriptional levels of genes in the collapsed status to those in the stable status. *^a^*: the values in reactors I and II on the left and right sides of the slash, respectively; *^b^*: from 2-hydroxymuconic semialdehyde to acetyl-CoA; *^c^*: from pyruvate to fructose 6-phosphate; *^d^*: from fructose 6-phosphate to pyruvate; *^e^*: from acetyl-CoA and oxaloacetate to 2-oxoglutarate; *^f^*: from 2-oxoglutarate to succinate; *^g^*: from succinate to oxaloacetate; *^h^*: from isocitrate to malate via glyoxylate; *^i^*: glutamate dehydrogenase [EC:1.4.1.4]; *^j^*: glutamate dehydrogenase [EC:1.4.1.3]; GDH: glutamate dehydrogenase; GS: glutamine synthetase; GOGAT: glutamate synthase. Arrow sizes are scaled to relative transcriptional levels: (1) bold arrow; highly expressed in both reactors I and II (*e.g.*, phenol/toluene 2-monooxygenase, catechol 2,3-dioxygenase, and GS), (2) middle-sized arrow; maintained or slightly decreased expression level (*e.g.*, 60%-100%) in either reactor, (3) dotted arrow; decreased (*e.g.*, less than 60%) in both reactors. Genes, locus tag numbers, enzymes, ko numbers, and fold values in # numbered pathways are shown in Table 1.

On the other hand, relative transcriptional levels in some metabolic pathways were moderately repressed, maintained, or increased. In the phenol-/catechol-degrading pathways, relative transcriptional levels decreased to 0.40±0.28-fold in the collapsed status, with the exception of genes encoding the subunit of PH (*dmpK*, CTR2_1592) and catechol 2,3-dioxygenase (*dmpB*, CTR2_1599). The genes encoding PH and catechol 2,3-dioxygenase were up-regulated by approximately 30- and 3.3-fold, respectively, in reactor I, and by 15- and 1.2-fold, respectively, in reactor II (Fig. 3A and 4, Table 1, and Supplementary Table S4-1).

In the TCA cycle, the relative transcriptional levels of genes encoding enzymes related to the conversion of 2-oxoglutarate (2-OG) to succinate (2-OG dehydrogenase [*sucA*, CTR2_2972, and *sucB*, CTR2_2971] and succinyl-CoA synthetase [*sucC*, CTR2_4841, and *sucD*, CTR2_4842]) significantly decreased by 0.34±0.065-fold in the collapsed status (Fig. 3B and 4), whereas those of the other genes moderately decreased by 0.73±0.25-fold from citrate to 2-OG (citrate synthase [*gltA*, CTR2_1354], aconitate hydratase [*acnA*, CTR2_1384, and *acnB*, CTR2_1366], and isocitrate dehydrogenase [*icd*, CTR2_1985]), and to 0.80±0.23-fold from succinate to oxaloacetate (succinate dehydrogenase/fumarate reductase [*sdhA/frdA*, CTR2_1357, *sdhB/frdB*, CTR2_1356, *sdhC/frdC*, CTR2_1359, and *sdhD/frdD*, CTR2_1358], fumarate hydratase [*fumA/fumB*, CTR2_5301, and *fumC*, CTR2_5299], and malate dehydrogenase [*mdh*, CTR2_1362, and *mqo*, CTR2_1070]) (Fig. 3B and 4, and Supplementary Table S4-2). In the glyoxylate shunt, the relative transcriptional levels of all 6 genes moderately decreased from the stable to collapsed status with the exception of the gene encoding isocitrate lyase (*aceA*, CTR2_1688) (Fig. 3C and 4, Table 1, and Supplementary Table S4-3). The relative transcriptional levels of the gene encoding isocitrate lyase (*aceA*, CTR2_1688) fluctuated in the collapsed status and became similar to or higher than those in the stable status.

The relative transcriptional levels of genes encoding enzymes in the biosynthesis of amino acids decreased to 0.63±0.12-fold in the collapsed status, with the exception of the synthesis of L-leucine, L-glutamate, and L-glutamine (Supplementary Fig. S7). The levels of glutamate dehydrogenase (GDH) (*gdhA*, CTR2_3645) in the collapsed status decreased to ~0.40-fold those in the stable status. The relative transcriptional levels of glutamate synthase (GOGAT) (*gltD*, CTR2_4073 and *gltB*, CTR2_4074) and glutamine synthetase (GS) (*glnA*, CTR2_1472) increased by approximately 2-fold in the collapsed status (Fig. 3D and 4, Table 1, and Supplementary Table S4-4).

In the urea cycle, the relative transcriptional levels of the 4 genes encoding enzymes were separated into two groups: the relative transcriptional levels of genes encoding enzymes related to the conversion of L-argininosuccinate to L-ornithine via L-arginine (argininosuccinate lyase [*argH*, CRT2_1809] and arginase [*rocF*, CRT2_0414]) moderately decreased to 0.82±0.22-fold in the collapsed status, whereas those of genes encoding enzymes related to the conversion from L-ornithine to L-arginino succinate via L-citrulline (ornithine carbamoyltransferase [*argF*, CTR2_1066] and argininosuccinate synthase [*argG*, CTR2_5281]) significantly decreased to 0.47±0.051-fold in the collapsed status (Fig. 3E and 4, Table 1, and Supplementary Table S4-5).

### Effects of amino acids on growth inhibition

**Fig. 5.**
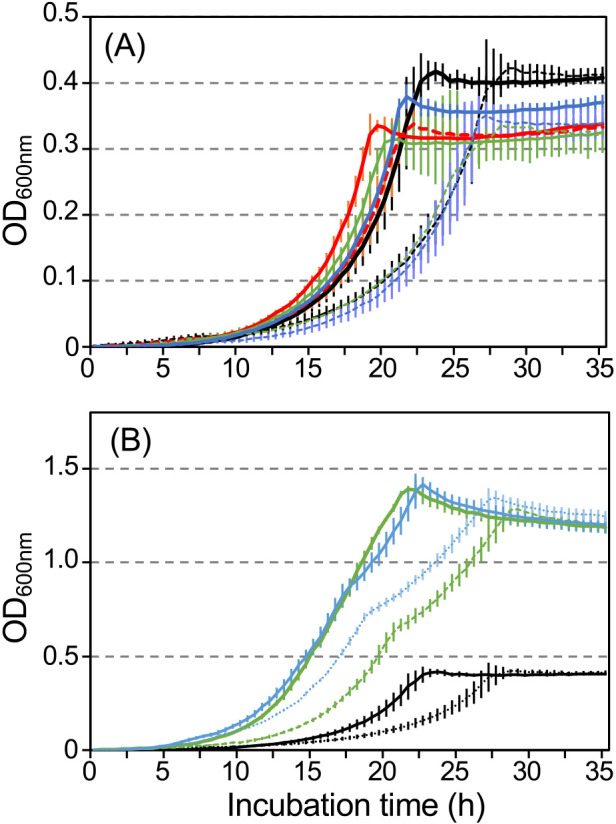
Effects of amino acids on the growth of strain R2. (A) Solid and broken lines show growth curves in the presence of phenol as a carbon source without and with the supernatant from the collapsed status, respectively. The black line shows the positive control, and the black solid line shows the negative control. Red line; L-arginine; green line: L-ornithine; and blue line: L-citrulline. (B) The black line shows the positive control, and the black solid line shows the negative control. Green line: L-glutamate, and blue line: L-glutamine.

We investigated whether the growth inhibition of strain R2 was rescued by amino acids from the urea cycle (L-arginine, L-ornithine, and L-citrulline), L-glutamate, and L-glutamine (Fig. 5). Lag times and *μ* values in the presence of phenol and amino acids from the urea cycle were similar to those in the positive control incubated in the presence of phenol only, whereas *OD*_max_ decreased to between 80 and 90% of the positive control (Table 2). Growth parameters in the presence of a supernatant (SN) collected from the collapsed culture were similar to those of the negative control, with the exception of L-arginine. The negative growth effect was 42±0.6% under control conditions, and 3.2±8.5, 34±11, and 40±15% in the presence of L-arginine, L-ornithine, and L-citrulline, respectively (Table 2). *OD*_max_ in the presence of L-glutamate and L-glutamine increased to ~3-fold that of control conditions and two-step growth was observed (Fig. 5B). Negative growth effects under L-glutamate, and L-glutamine conditions were 59±0.36 and 27±10%, respectively (Table 2), even when a higher *μ* value in two-step growth was used for the calculation. Phenol concentrations were below the detection limit under all conditions after growth reached a plateau.

## Discussion

The present study attempted to elucidate the collapsing mechanism induced by feedback growth inhibition in strain R2. Collapse was not observed under batch conditions, it only occurred under chemostat conditions. The results obtained revealed that growth inhibition and metabolic changes had already occurred before the collapse, which was attributed to negative feedback induced by the accumulation of hydrophilic metabolites produced from strain R2. Unexpectedly, increases were noted in the relative transcriptional levels of the genes encoding PH and catechol 2,3-dioxygenase (Fig. 4, Table 1). These genes are located in an operon regulated by the regulator protein DmpR (Azwani *et al.*, 2017, 2021), which accelerates transcription by binding with phenol and its dissociation constant is 16‍ ‍μM (O’Neill *et al.*, 1998), indicating that phenol concentrations had increased in cells. Since PH and catechol 2,3-dioxygenase were competitively inhibited by the metabolites (Fig. 2), we considered a deficiency in a usable carbon source to be a trigger for collapse. Transcriptomic analyses showed that the actual condition of the collapsing mechanism was metabolically complex; metabolites induced an imbalance in carbon and nitrogen metabolism in cells, which resulted in collapse. We discussed how the imbalance occurred and why L-arginine effectively rescued it.

The transcriptomic analysis demonstrated that relative transcriptional levels decreased in the majority of carbon metabolic pathways, indicating that the supply of carbon skeletons for nitrogen assimilation was limited under growth-inhibiting conditions. On the other hand, the relative transcriptional levels of some metabolic pathways moderately decreased, were maintained, increased, or fluctuated, which were considered to be adaptive processes to negative feedback. The relative transcriptional level of the isocitrate lyase gene (*aceA*, CTR2_1688) related to the TCA cycle and glyoxylate shunt fluctuated and increased under collapsed conditions (Fig. 3C), indicating that strain R2 adapted to a carbon source deficiency because carbon was not released as CO_2_ in the glyoxylate shunt. The relative transcriptional levels of the metabolic pathways from citrate to 2-OG in the TCA cycle in the collapsed state were limited to approximately 0.70-fold those in the stable status, whereas the metabolic pathways from 2-OG to succinate were repressed to approximately 0.35-fold (Fig. 3B and 4, and Supplementary Table S4-2). 2-OG may be used in metabolic pathways other than the TCA cycle because it plays a major role in carbon skeletons for the biosynthesis of nitrogenous compounds via L-glutamate and L-glutamine (Merrick and Edwards, 1995). GDH and GS have been widely suggested to play a central role in responses to ammonia stress by converting ammonia to non-toxic or less toxic nitrogenous compounds (Zhang *et al.*, 2020). In the collapsed status, the transcriptional level of GDH (*gdhA*, CTR2_3645) decreased (Fig. 3D and 4, and Table 1), whereas those of GS (*glnA*, CTR2_1472) and GOGAT (*gltD*, CTR2_4073 and *gltB*, CTR2_4074) (the GS-GOGAT pathway) increased (Fig. 3D and 4, and Table 1). Differences in activities between the GS-GOGAT and GDH pathways are considered to depend on differences in the affinity for ammonium of GS (~0.1‍ ‍mM) (Sharkey and Engel, 2008) and GDH (more than 1‍ ‍mM) (Alibhai and Villafranca, 1994), whereas the GS-GOGAT pathway is the main ammonium-assimilatory route even with a high ammonium concentration (10‍ ‍mM) in *E. coli* (Yuan *et al.*, 2009). The *K*_m_ values of GS and GDH for 2-OG are ~0.24‍ ‍mM (Mantsala and Zalkin, 1976) and ~0.64‍ ‍mM (Veronese *et al.*, 1975; Sharkey and Engel, 2008), respectively, suggesting that strain R2 is under 2-OG deficiency conditions. The activity of Icd (CTR2_1985) producing 2-OG from isocitrate in the TCA cycle may be repressed because enzyme activity is positively regulated by metabolites derived from glycolysis/gluconeogenesis (Cozzone and El-Mansi, 2005), which supports the hypothesis of a 2-OG pool deficiency in strain R2 in the collapsed state.

2-OG acts as a metabolic signal of small and transient fluctuations in ammonium availability in *Escherichia coli*, *i.e.*, the concentration of 2-OG increases with ammonium limitations under steady-state growth conditions (Senior, 1975; Reyes-Ramirez *et al.*, 2001) and *vice versa* (Yuan *et al.*, 2009; Radchenko *et al.*, 2010; Yan *et al.*, 2011). A negative correlation has been reported between ammonium availability and 2-OG accumulation in other microorganisms (Muro-Pastor *et al.*, 2001; Dodsworth *et al.*, 2005; Brauer *et al.*, 2006). This negative correlation contributes to the balance between carbon and nitrogen metabolism: the decrease in 2-OG and increase in L-glutamine facilitate the construction of the complex of the ammonium transporter (AmtB) and regulator protein (GlnK), resulting in the repression of NH_4_^+^ influx through the AmtB-GlnK complex (Radchenko *et al.*, 2010), and the replenishment of the 2-OG pool is then achieved by increased transamination after the consumption of ammonium. However, the relative transcriptional levels of nitrogenous compound synthesis decreased not only in nucleotide synthesis (Supplementary Fig. S6), but also in amino acid synthesis, with the exception of L-leucine, L-glutamate, and L-glutamine (Supplementary Fig. S7). Furthermore, the gene encoding the ammonium transporter Amt family (CTR2_4687) was up-regulated by ~190-fold in the collapsed status (Supplementary Table S5-1), suggesting that cellular ammonium concentrations increased in the collapsed status. In parallel, the genes encoding nitrite reductase (*nirB*, CTR2_4180) and the urea ABC transporter (CTR2_0055) were up-regulated by ~155- and ~20-fold, respectively, in the collapsed status (Supplementary Table S5-1), indicating that strain R2 attempted to decrease endogenous ammonium toxicity and balance carbon and nitrogen metabolism by reducing excess ammonium. The gene encoding AmtB was significantly up-regulated even under conditions of low 2-OG and high glutamine concentrations, which has yet to be resolved with the identification of growth-inhibiting metabolites. This imbalance may affect various metabolic pathways; *e.g.*, outer membrane protein porin (CTR2_5017) and cell division protein (CTR2_0482) were down-regulated (Supplementary Table S5-3 and S5-4), whereas PilA (CTR2_4856) was up-regulated in the collapsed status (Supplementary Table S5-1 and S5-2). The imbalance in metabolism related to the cell structure may have caused the disordered cells observed in the collapsed status (Supplementary Fig. S2B). The mechanisms maintaining the balance between carbon and nitrogen metabolism in strain R2 have not yet been elucidated.

The urea cycle is an essential pathway for the disposal of ammonia in mammals, and the genes encoding enzymes in the urea cycle in mammals are evolutionarily derived from bacterial genes for the detoxification of ammonia (Walsh and Patricia, 1995). A previous study reported that the urea cycle of *Helicobacter pylori* acts as an effective mechanism to extrude excess nitrogen from cells (Mendz and Hazell, 1996). Therefore, we attempted to enhance the activity of the urea cycle by adding amino acids to the urea cycle because the urea cycle of strain R2 was repressed in the collapsed status (Fig. 3E and 4, and Table 1). Only L-arginine enabled the rescue of strain R2 from growth inhibition without an increase in the number of cells (Fig. 5A and Table 2), indicating that L-arginine was not used in anabolic processes, but functioned as a trigger to enhance the urea cycle. The mechanisms by which L-arginine rescues strain R2 from growth inhibition warrant further study.

## Conclusion

The present study revealed the metabolic responses of strain R2 to negative feedback (Fig. 4 and Table 1). The imbalance in carbon and nitrogen metabolism was caused by both an increase in the concentration of NH_4_^+^ and a decrease in the supply of carbon skeletons, which resulted in collapse. 2-OG and PRPP are at the metabolic intersection between carbon and nitrogen metabolism, with 2-OG playing a major role as not only a master regulator (Huergo and Dixon, 2015), but also a major carbon skeleton in nitrogen-assimilatory reactions (Commichau *et al.*, 2006). Nitrogen regulates primary and secondary metabolism in various bacteria (Merrick and Edwards, 1995); therefore, a more detailed understanding of the regulatory mechanisms of NH_4_^+^ concentrations in cells will be indispensable for managing microorganisms. The monitoring of these compound concentrations in cells will provide insights into collapse and rescue mechanisms and these analyses will contribute to the clarification of bacterial coexisting mechanisms in microbial ecosystems.

## Citation

Mohd Din, A. R. J., Suzuki, K., Honjo, M., Amano, K., Nishimura, T., Moriuchi, R., et al. (2021) Imbalance in Carbon and Nitrogen Metabolism in *Comamonas testosteroni* R2 Is Caused by Negative Feedback and Rescued by L-arginine. *Microbes Environ ***36**: ME21050.

https://doi.org/10.1264/jsme2.ME21050

## Supplementary Material

Supplementary Material

## Figures and Tables

**Table 1.** Lists of genes encoding pathways related to the collapsed status.

Metabolism	No*^a^*	Gene	Locus tag number	Enzyme	Substrates	Products	ko number	Fold change in reactor I*^b^*	Fold change in reactor II*^c^*
Phenol degradation	1	*dmpK*	CTR2_1592	phenol/toluene 2-monooxygenase (NADH) P0/A0	Phenol	catechol	K16249	31	15
Catechol degradation	2	*dmpB*	CTR2_1599	catechol 2,3-dioxygenase [EC:1.13.11.2]	catechol	2-Hydroxymuconic semialdehyde	K00446	3.3	1.2
PRPP synthesis	3	*prsA*	CTR2_3950	ribose-phosphate pyrophosphokinase [EC:2.7.6.1]	D-Ribose 5-phosphate	PRPP	K00948	0.44	0.47
TCA cycle	4	*gltA*	CTR2_1354	citrate synthase [EC:2.3.3.1]	Acetyl-CoA, Oxaloacetate	Citrate	K01647	0.90±0.24	0.53±0.21
		*acnA*	CTR2_1384	aconitate hydratase [EC:4.2.1.3]	Citrate	Isocitrate	K01681		
		*acnB*	CTR2_1366	aconitate hydratase 2/2-methylisocitrate dehydratase [EC:4.2.1.3 4.2.1.99]			K01682		
	5	*icd*	CTR2_1985	isocitrate dehydrogenase [EC:1.1.1.42]	Isocitrate	2-oxoglutarate	K00031	0.72	0.85
Glyoxylate shunt	6	*aceA*	CTR2_1688	isocitrate lyase [EC:4.1.3.1]	Isocitrate	Glyoxylate	K01637	0.83	2.1
	7	*aceB*	CTR2_0005	malate synthase [EC:2.3.3.9]	Glyoxylate	Malate	K01638	0.99	0.78
GDH	8	*gdhA*	CTR2_3645	glutamate dehydrogenase (NADP^+^) [EC:1.4.1.4]	2-OG, NH_3_, NADPH	L-glutamate, NADP^+^	K00262	0.34	0.44
	9	*gdhA*	CTR2_4700	glutamate dehydrogenase (NAD^+^) [EC:1.4.1.3]	L-glutamate, NAD^+^	2-OG, NH_3_, NADH	K00261	2.8	0.49
GOGAT	10	*gltD*	CTR2_4073	glutamate synthase (NADPH) small chain [EC:1.4.1.13]	L-glutamine, 2-OG, NADPH	L-glutamate	K00266	2.0	0.68
		*gltB*	CTR2_4074	glutamate synthase (NADPH) large chain [EC:1.4.1.13]	L-glutamine, 2-OG, NADPH	L-glutamate	K00265	2.3	1.7
GS	11	*glnA*	CTR2_1472	glutamine synthetase [EC:6.3.1.2]	L-glutamate, NH_3_, ATP	L-glutamine	K01915	2.0	2.6
Urea cycle	12	*argF*	CTR2_1066	ornithine carbamoyltransferase [EC:2.1.3.3]	L-ornithine	L-citrulline	K00611	0.53	0.51
	13	*argG*	CTR2_5281	argininosuccinate synthase [EC:6.3.4.5]	L-citrulline, L-aspartate	L-argininosuccinate	K01940	0.45	0.42
	14	*argH*	CTR2_1809	argininosuccinate lyase [EC:4.3.2.1]	L-argininosuccinate	L-arginine, fumarate	K01755	0.74	1.1
	15	*rocF*	CTR2_0414	arginase [EC:3.5.3.1]	L-arginine	L-ornithine, Urea	K01476	0.83	0.59
Nucleotide metabolism	16	*purF*	CTR2_5274	amidophosphoribosyltransferase [EC:2.4.2.14]	L-glutamine, PRPP	Ribosylamine-5P	K00764	0.35	0.48

*^a^*; The number corresponds to “#number” shown in Fig. 4 *^b^*; TPM value at I-5 divided by TPM value at I-1. *^c^*; TPM value at II-5 divided by TPM value at II-1.

**Table 2.** Effects of amino acids on the growth of strain R2

Condition*^a^*	Lag time (h)	Growth rate constant (*μ*)	OD_600‍ ‍nm_	Specific growth activity (U)	Negative growth effect (%)*^h^*
Positive control	10±1.2	0.25±0.0064	0.40±0.0093	0.048±0.0027	42±0.60
Negative control	12±1.0	0.17±0.011	0.40±0.0087	0.027±0.0024	
Arg*^b^*	10±0.50	0.29±0.0074	0.32±0.0064	0.044±0.00070	3.2±8.5
Arg+SN*^c^*	8.8±0.58	0.25±0.013	0.32±0.019	0.043±0.0037	
Orn*^d^*	9.5±1.0	0.26±0.0065	0.31±0.039	0.041±0.0050	34±11
Orn+SN	11±0.58	0.19±0.031	0.33±0.029	0.027±0.0030	
Ctr*^e^*	11±0.29	0.26±0.0057	0.36±0.010	0.041±0.0022	40±15
Ctr+SN	11±0.29	0.22±0.0090	0.27±0.083	0.025±0.00061	
Glu*^f^*	4.8±0.29	0.35±0.0093	1.2±0.036	0.41±0.015	59±6.3
Glu+SN	8.5±1.5	0.25±0.0047	1.2±0.035	0.17±0.025	
Gln*^g^*	9.0±1.0	0.26±0.0040	1.2±0.046	0.17±0.021	27±10
Gln+SN	11±0.56	0.22±0.0051	1.3±0.027	0.12±0.0033	

*^a^*; Phenol was added as the sole carbon and energy source in all conditions. *^b^*; Arg: L-arginine, *^c^*; SN: supernatant collected from collapsed chemostat cultures. *^d^*; Orn: L-ornithine, *^e^*; Ctr: L-citrulline, *^f^*;‍ ‍Glu: L-glutamate, *^g^*; Gln: L-glutamine, *^h^*; negative growth effect (%): the effect was calculated by the following equation: (1–*U_amino acid_*⁄*U_amino acid_*_+_*_SN_*)×100, where *U*_amino acid_ is relative growth activity in the presence of phenol and an amino acid, and U_amino acid+SN_ is relative growth activity in the presence of phenol, the amino acid, and the supernatant.
