# Supplementary material for: Imbalance in Carbon and Nitrogen Metabolism in Comamonas testosteroni R2 Is Caused by Negative Feedback and Rescued by L-arginine
Source: Microbes Environ. 2021 Oct 13;36(4):ME21050. doi: 10.1264/jsme2.ME21050 (PMC8674442; doi:10.1264/jsme2.ME21050)
Supplement: Supplementary file 1 — Supplementary Material [file 36_21050_s1.pdf]

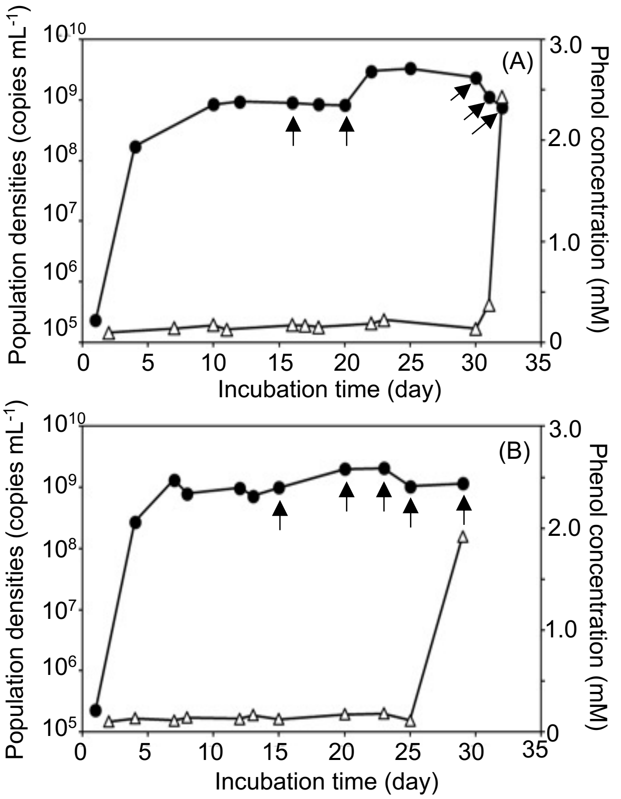

Supplementary Figure S1.

Growth and phenol concentration of strain R2 in chemostat cultures for transcriptomic analysis.

(A) Reactor I. Cells were collected at day 17 (sample I-1), day 22 (sample I-2), day 30 (sample I-3), day 31 (sample I-4), and day 32 (sample I-5).

(B) Reactor II. Cells were collected at day 15 (sample II-1), day 20 (sample II-2), day 23 (sample II-3), day 25 (sample II-4), and day 28 (sample II-5). Arrows indicate sampling date.

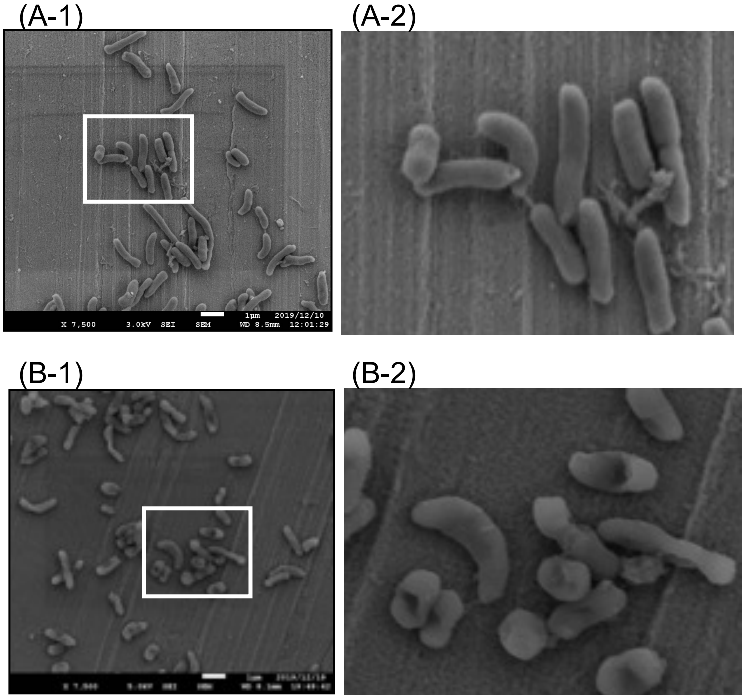

Supplementary Figure S2. SEM observation of strain R2 cells on day 18 (A) and day 38 (B) in the chemostat culture. The scale bar indicates 1 μm. The area shown in a white square in A-1 and B-1 was shown as a enlarged figure in A-2 and B-2, respectively.

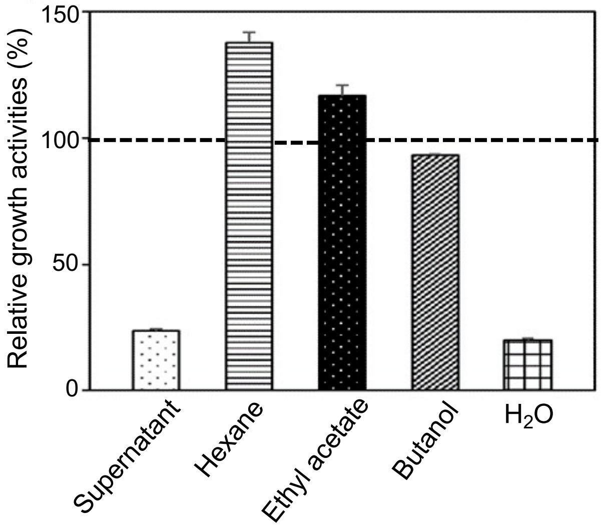

Supplementary Figure S3.
A supernatant collected from chemostat cultures at the collapsed status was fractioned by using hexane, ethyl acetate, butanol, and H_2O. Fractions with hexane, ethyl acetate, and butanol were evaporated and resuspended in H_2O. Effects of these and the H_2O fraction on the growth of strain R2 were evaluated, indicating that the H_2O fraction showed similar the growth inhibition to the supernatant.

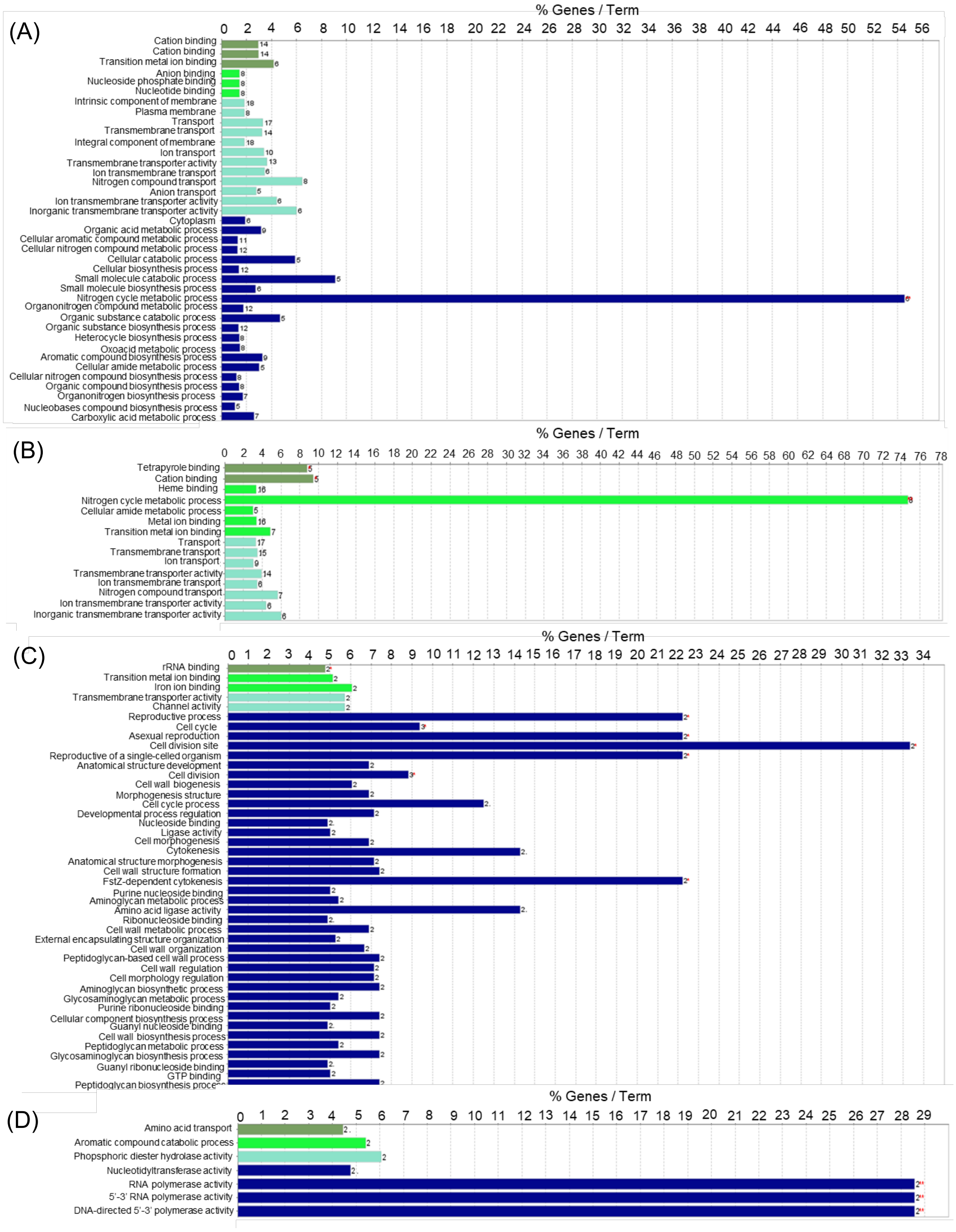

Supplementary Figure S4. Functional grouping of up- and down-regulated genes in the reactors I and II by ClueGo analysis. (A) Up-regulated genes in the reactor I, (B) Up-regulated genes in the reactor II, (C) Down-regulated genes in the reactor I, (D) Down-regulated genes in the reactor II. The bars represent the percentage of differentially expressed genes associated with the terms. The number of differentially expressed genes per term is shown as bar label. *Sign referred to mid-P values of two-sided (enrichment/depletion) tests based on the hyper geometric distribution.

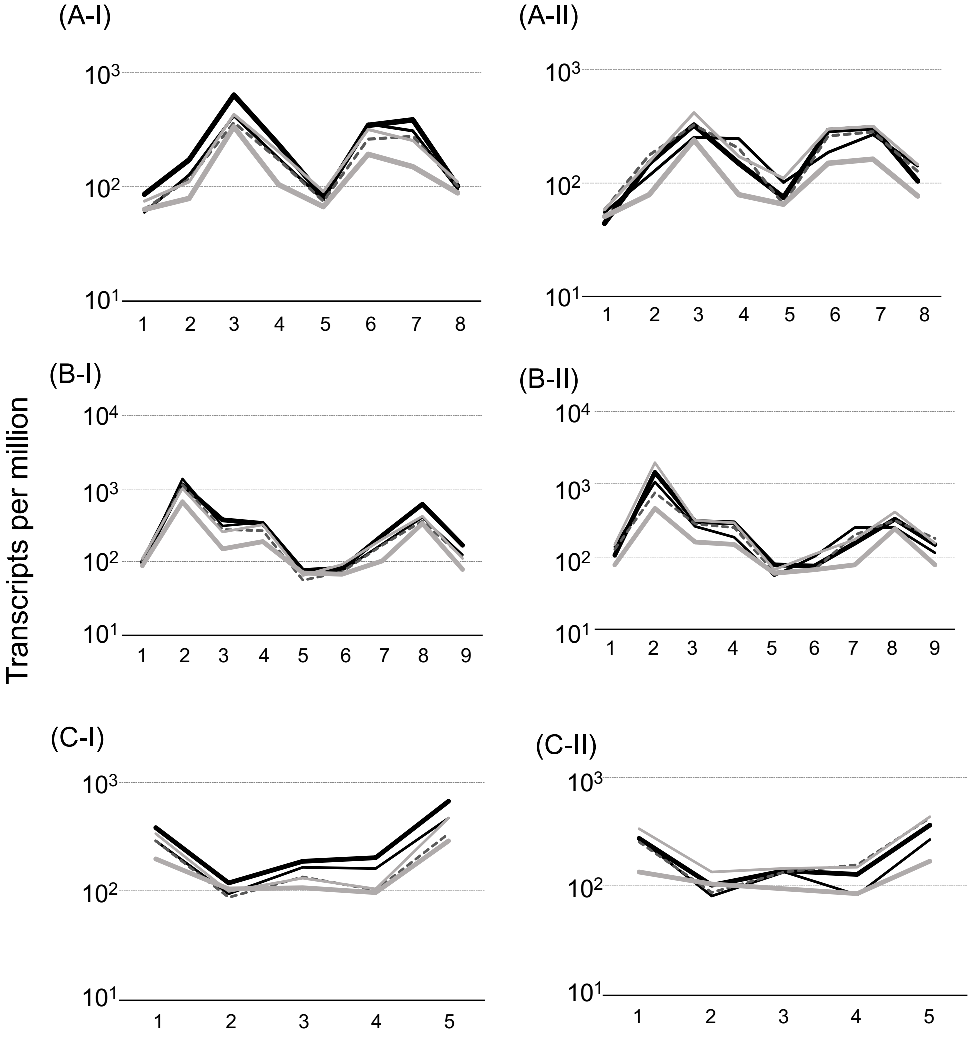

Supplementary Figure S5.
Relatively transcriptional levels of genes encoding enzymes in (A) glycolysis, (B) gluconeogenesis, and (C) pentose phosphate and PRPP synthesis. (A-I), (B-I), (C-I) in the reactor I, and (A-II), (B-II), (C-II) in the reactor II. Bold black line: stage I-1 or stage II-1; black line: stage I-2 or stage II-2; broken line: stage I-3 or stage II-3; gray line: stage I-4 or stage II-4; and bold gray line: stage I-5 or II-5. The genes, locus-tag number, enzymes, substrates and products, ko number, and transcripts per million values of the genes were listed on Supplementary Tables S2.

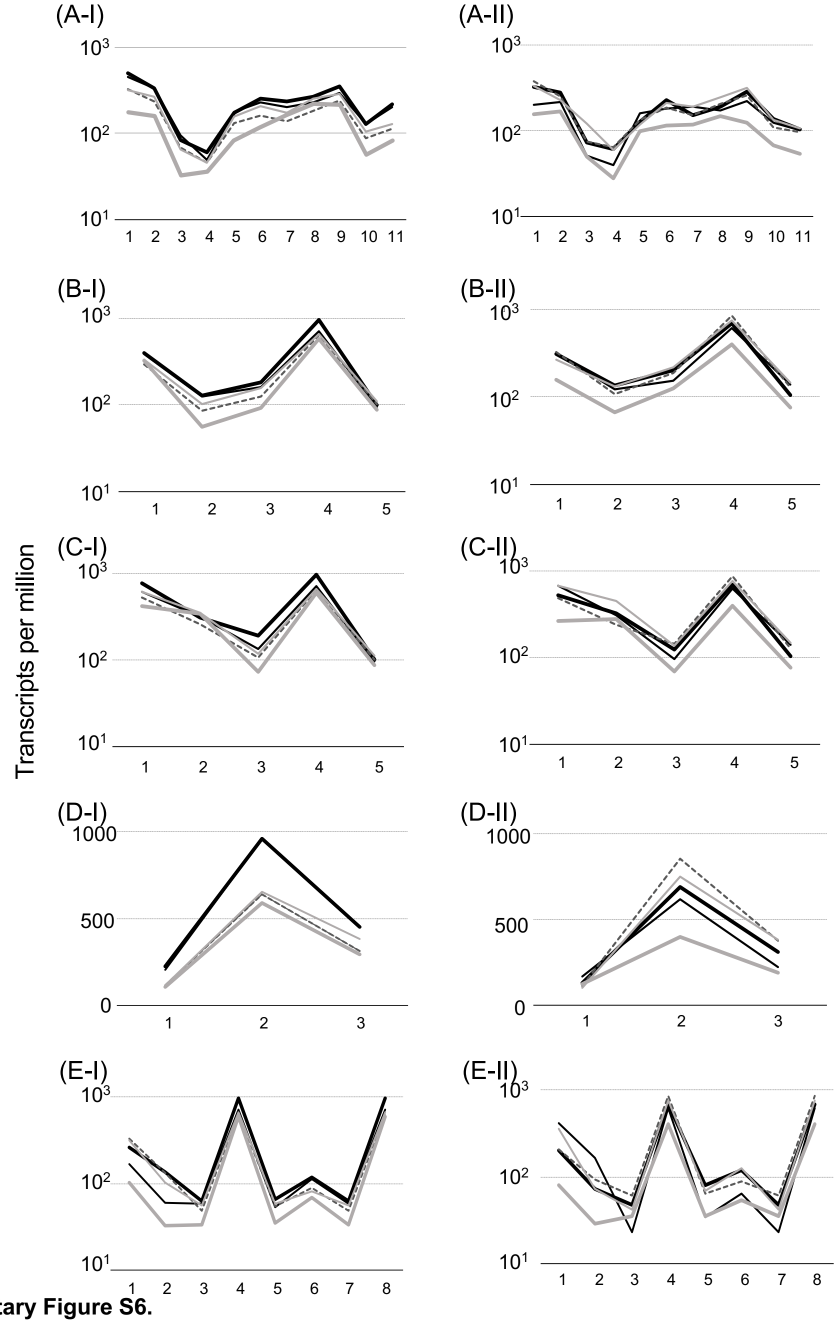

Supplementary Figure S6.

Relatively transcriptional levels of genes encoding enzymes in (A) Inosine monophosphate synthesis, (B) Adenine ribonucleotide synthesis, (C) Guanine ribonucleotide synthesis, (D) Pyrimidine ribonucleotide biosynthesis, and (E) Pyrimidine deoxyribonucleotide biosynthesis (UDP to dTTP). (A-I), (B-I), (C-I), (D-I), and (E-I) in the reactor I, and (A-II), (B-II), (C-II)), (D-II), and (E-II) in the reactor II. Bold black line: stage I-1 or stage II-1; black line: stage I-2 or stage II-2; broken line: stage I-3 or stage II-3; gray line: stage I-4 or stage II-4; and bold gray line: stage I-5 or II-5. The genes, locus-tag number, enzymes, substrates, products, ko number, and transcripts per million values of the genes were listed on Supplementary Tables S3.

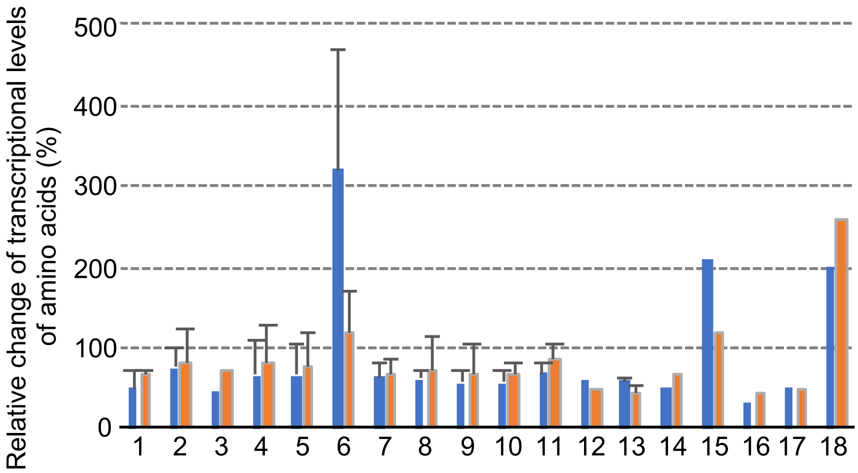

Supplementary Figure S7.
Relative change of transcriptional levels of genes encoding amino acids at the collapsed status compared with the stable status.
Blue bars: reactor I; red bars: reactor II. The value was calculated using the following equation: (Transcripts per million [TPM] of an amino acid in I-5 per TPM of the amino acids in I-1 in the reactor I) × 100, which was the same as in reactor II. 1: L-serine; 2: L-threonine; 3: L-cysteine; 4: L-Valine/L-isoleucine; 5: L-isoleucine; 6: L-leucine; 7: L-lysine; 8: L-ornithine; 9: L-arginine; 10: L-proline; 11: Skimate; 12: L-phenylalanine; 13: L-tyrosine; 14: L-glutathione; 15: L-glutamate (by glutamate synthase); 16: L-glutamate (by glutamate dehydrogenase [EC:1.4.1.4]); 17: L-glutamate (by glutamate dehydrogenase [EC:1.4.1.3]); 18: L-glutamine (by glutamine synthetase [EC:6.3.1.2]). The genes, locus-tag number, enzymes, substrates, products, ko number, and transcripts per million values of the genes were listed on Supplementary Table S3.

Table S1. Summary of RNA-Seq data analyzed in this study

Sample	Number of raw reads	Total base of raw reads (bp)	Number of filtered reads	Total base of filtered reads (bp)	Number of mapped reads	Mapping rate (%)	Accession number
I-1	50,570,842	5,107,655,042	49,108,684	4,907,610,374	47,373,209	96.47	DRR309243
I-2	56,204,212	5,676,625,412	54,553,096	5,451,667,863	51,898,264	95.13	DRR309244
I-3	52,757,580	5,328,515,580	51,114,262	5,107,604,355	47,907,487	93.73	DRR309245
I-4	48,317,836	4,880,101,436	46,728,274	4,669,418,429	42,812,014	91.62	DRR309246
I-5	46,658,848	4,712,543,648	45,238,006	4,521,042,057	42,264,870	93.43	DRR309247
II-1	42,982,722	4,341,254,922	41,425,540	4,139,089,785	30,251,476	73.03	DRR309248
II-2	45,751,028	4,620,853,828	43,970,598	4,391,210,858	33,030,978	75.12	DRR309249
II-3	46,626,246	4,709,250,846	44,143,672	4,404,519,514	25,657,249	58.12	DRR309250
II-4	46,023,700	4,648,393,700	43,707,376	4,362,946,285	19,153,223	43.82	DRR309251
II-5	48,629,992	4,911,629,192	46,229,750	4,614,444,293	18,892,478	40.87	DRR309252

Table S2-1 Glycolysis

Glycolysis						
No.	gene	locus-tag	Enzymes	Substrates	Products	ko number
1	*pgi*	CTR2_4084	glucose-6-phosphate isomerase [EC:5.3.1.9]	D-glucose 6-phosphate	D-fructose 6-phosphate	K01810
2	*fbp*	CTR2_1715	fructose-1,6-bisphosphatase I [EC:3.1.3.11]	D-fructose 6-phosphate	D-fructose 1,6-bisphosphate	K03841
3	*fbaA*	CTR2_4997	fructose-bisphosphate aldolase, class II [EC:4.1.2.13]	D-fructose 1,6-bisphosphate	D-glyceraldehyde 3-phosphate	K01624
4	*gapA*	CTR2_4984	glyceraldehyde 3-phosphate dehydrogenase [EC:1.2.1.12]	D-glyceraldehyde 3-phosphate	3-phospho-D-glyceroyl phosphate	K00134
5	*pgk*	CTR2_4891	phosphoglycerate kinase [EC:2.7.2.3]	3-phospho-D-glyceroyl phosphate	3-phospho-D-glycerate	K00927
6	*gpmA*	CTR2_0716	2,3-bisphosphoglycerate-dependent phosphoglycerate mutase [EC:5.4.2.11]	3-phospho-D-glycerate	2-phospho-D-glycerate	K01834
7	*eno*	CTR2_3617	enolase [EC:4.2.1.11]	2-phospho-D-glycerate	phosphoenolpyruvate	K01689
8	*pyk*	CTR2_4991	pyruvate kinase [EC:2.7.1.40]	phosphoenolpyruvate	pyruvate	K00873

Transcripts per million values of target genes in Reactor I									
No.	I-1	I-2	I-3	I-4	I-5	log_2FC	logCPM	FDR	Fold[a]
1	85.236	60.244	62.999	73.806	63.254	0.017933	7.37745312	0.99964082	0.74
2	170.472	124.958	114.613	111.344	78.487	-0.6707331	7.4226224	0.97657824	0.46
3	622.105	410.593	356.286	420.234	328.676	-0.4722521	9.42659846	0.99964082	0.53
4	230.653	175.174	169.686	203.457	103.61	-0.706302	7.81050185	0.95390773	0.45
5	81.743	75.864	74.242	90.473	66.754	0.15598676	6.96168288	0.99964082	0.82
6	336.467	346.948	261.23	315.338	191.057	-0.3682164	8.06755294	0.99964082	0.57
7	377.119	307.639	271.065	253.15	149.775	-0.8839672	8.81957928	0.82846204	0.40
8	100.742	95.336	102.348	110.515	87.417	0.24353155	7.57660787	0.99964082	0.87

[a]; Fold was calculated using the equation as follows:

Transcripts per million [TMP] values of I-5 / TMP value of I-1

Transcripts per million values of target genes in Reactor II									
No.	II-1	II-2	II-3	II-4	II-5	log_2FC	logCPM	FDR	Fold[b]
1	44.293	55.112	58.017	58.605	51.391	0.52905448	6.76359284	0.99774764	1.16
2	148.171	114.774	178.003	154.253	78.683	-0.5983729	7.30006008	0.99774764	0.53
3	325.744	253.981	320.216	409.879	240.811	-0.1211373	8.70123793	0.99774764	0.74
4	150.703	247.896	200.958	170.472	78.317	-0.6295432	7.27803898	0.99774764	0.52
5	73.961	101.119	65.593	110.498	65.415	0.1375476	6.85899901	0.99774764	0.88
6	290.899	183.269	255.009	293.111	147.936	-0.6607929	7.78846205	0.99614483	0.51
7	307.853	265.136	278.837	314.751	160.222	-0.6274482	8.67265989	0.99774764	0.52
8	104.768	138.964	126.976	146.159	76.686	-0.1354671	7.49139871	0.99774764	0.73

[b]; Fold was calculated using the equation as follows:

Transcripts per million [TMP] values of II-5 / TMP value of II-1

Table S2-2 Gluconeogenesis

Gluconeogenesis						
No.	gene	locus-tag	Enzymes	Substrates	Products	ko number
1	*pyk*	CTR2_4991	pyruvate kinase [EC:2.7.1.40]	Pyruvate	Phosphoenolpryruvate	K00873
2	*pckA*	CTR2_4753	phosphoenolpyruvate carboxykinase (GTP) [EC:4.1.1.32]	Oxaloacetate	Phosphoenolpryruvate	K01596
3	*eno*	CTR2_3617	enolase [EC:4.2.1.11]	Phosphoenolpryruvate	2-phospho-D-glycerate	K01689
4	*qpmA*	CTR2_0716	2,3-bisphosphoglycerate-dependent phosphoglycerate mutase [EC:5.4.2.11]	2-phospho-D-glycerate	3-phospho-D-glycerate	K01834
5	*qpmB*	CTR2_0308	2,3-bisphosphoglycerate-dependent phosphoglycerate mutase [EC:5.4.2.11]			K15634
6	*pgk*	CTR2_4891	phosphoglycerate kinase [EC:2.7.2.3]	3-phospho-D-glycerate	D-glycerate 1,3-diphosphate	K00927
7	*gapA*	CTR2_4984	glyceraldehyde 3-phosphate dehydrogenase [EC:1.2.1.12]	D-glycerate 1,3-diphosphate	D-glyceraldehyde 3-phosphate	K00134
8	*fbaA*	CTR2_4997	fructose-bisphosphate aldolase, class II [EC:4.1.2.13]	D-glyceraldehyde 3-phosphate	fructose 1,6-bisphosphate	K01624
9	*fbp*	CTR2_1715	fructose-1,6-bisphosphatase I [EC:3.1.3.11]	fructose 1,6-bisphosphate	fructose 6-phosphate	K03841

Transcripts per million values of target genes in Reactor I									
No.	I-1	I-2	I-3	I-4	I-5	log_2FC	logCPM	FDR	Fold[a]
1	100.742	95.336	102.348	110.515	87.417	0.24353155	7.57660787	0.99964082	0.87
2	1110.93	1360.306	1113.063	1013.227	649.437	-0.326272	11.1278914	0.99964082	0.58
3	377.119	307.639	271.065	253.15	149.775	-0.8839672	8.81957928	0.82846204	0.40
4	336.467	346.948	261.23	315.338	191.057	-0.3682164	8.06755294	0.99964082	0.57
5	75.164	69.21	55.596	65.186	70.486	0.3554599	6.054899	0.99964082	0.94
6	81.743	75.864	74.242	90.473	66.754	0.15598676	6.96168288	0.99964082	0.82
7	230.653	175.174	169.686	203.457	103.61	-0.706302	7.81050185	0.95390773	0.45
8	622.105	410.593	356.286	420.234	328.676	-0.4722521	9.42659846	0.99964082	0.53
9	170.472	124.958	114.613	111.344	78.487	-0.6707331	7.4226224	0.97657824	0.46

[a]; Fold was calculated using the equation as follows:

Transcripts per million [TMP] values of I-5 / TMP value of I-1

Transcripts per million values of target genes in Reactor II									
No.	II-1	II-2	II-3	II-4	II-5	log_2FC	logCPM	FDR	Fold[a]
1	104.768	138.964	126.976	146.159	76.686	-0.1354671	7.49139871	0.99774764	0.73
2	1414.605	1078.012	763.491	1987.38	462.95	-1.2967658	11.1732099	0.51123257	0.33
3	307.853	265.136	278.837	314.751	160.222	-0.6274482	8.67265989	0.99774764	0.52
4	290.899	183.269	255.009	293.111	147.936	-0.6607929	7.78846205	0.99614483	0.51
5	77.635	54.095	61.711	66.895	59.901	-0.0594228	5.93750882	0.99774764	0.77
6	73.961	101.119	65.593	110.498	65.415	0.1375476	6.85899901	0.99774764	0.88
7	150.703	247.896	200.958	170.472	78.317	-0.6295432	7.27803898	0.99774764	0.52
8	325.744	253.981	320.216	409.879	240.811	-0.1211373	8.70123793	0.99774764	0.74
9	148.171	114.774	178.003	154.253	78.683	-0.5983729	7.30006008	0.99774764	0.53

[b]; Fold was calculated using the equation as follows:

Transcripts per million [TMP] values of II-5 / TMP value of II-1

Table S2-3 Pentose phosphate and PRPP pathways

Pentose phosphate and PRPP pathways						
No.	gene	locus-tag	Enzymes	Substrates	Products	ko number
1	*tktA, tktB*	CTR2_4985	transketolase [EC:2.2.1.1]	sedoheptulose 7-phosphate / D-glyceraldehyde 3-phosphate	D-ribose 5-phosphate/D-xylulose 5-phosphate	K00615
2	*talA, talB*	CTR2_4083	transaldolase [EC:2.2.1.2]	sedoheptulose 7-phosphate / D-glyceraldehyde 3-phosphate	D-erythrose 4-phosphate/D-fructose 6-phosphate	K00616
3	*rpe*	CTR2_0102	ribulose-phosphate 3-epimerase [EC:5.1.3.1]	D-Ribulose 5-phosphate	D-xylulose 5-phosphate	K01783
4	*rpiA*	CTR2_2754	ribose 5-phosphate isomerase A [EC:5.3.1.6]	D-Ribose 5-phosphate	D-ribulose 5-phosphate	K01807
5	*prsA*	CTR2_3950	ribose-phosphate pyrophosphokinase [EC:2.7.6.1]	D-Ribose 5-phosphate	PRPP	K00948

Transcripts per million values of target genes in Reactor I									
No.	I-1	I-2	I-3	I-4	I-5	log_2FC	logCPM	FDR	Fold[a]
1	379.982	286.642	285.658	339.591	200.018	-0.4775613	9.65715077	0.99964082	0.53
2	118.506	94.183	86.974	100.225	105.127	0.27539633	7.2644199	0.99964082	0.89
3	187.869	164.494	134.334	130.525	108.95	-0.337798	7.11349403	0.99964082	0.58
4	205.003	160.488	101.566	105.557	96.266	-0.6422683	7.15071561	0.98528691	0.47
5	667.188	464.351	338.39	469.86	290.782	-0.7499098	9.26803301	0.92756493	0.44

[a]; Fold was calculated using the equation as follows:

Transcripts per million [TMP] values of I-5 / TMP value of I-1

Transcripts per million values of target genes in Reactor II									
No	II-1	II-2	II-3	II-4	II-5	log_2FC	logCPM	FDR	Fold[b]
1	272.778	277.643	253.694	332.82	136.232	-0.6869447	9.14561962	0.99009804	0.50
2	101.092	81.95	87.193	136.371	105.05	0.3700476	7.13855037	0.99774764	1.0
3	138.375	134.423	134.981	145.029	93.663	-0.2482988	6.7645779	0.99774764	0.68
4	128.596	84.362	155.57	148.2	84.611	-0.2891893	6.67829686	0.99774764	0.66
5	361.866	266.282	426.096	429.312	170.18	-0.7736579	8.42762625	0.96412124	0.47

[b]; Fold was calculated using the equation as follows:

Transcripts per million [TMP] values of II-5 / TMP value of II-1

Table S3-1 Inosine monophosphate synthesis

Inosine monophosphate synthesis						
No.	gene	locus-tag	Enzymes	Substrates	Products	ko number
1	*purF*	CTR2_5274	amidophosphoribosyltransferase [EC:2.4.2.14]	L-glutamine & PRPP	Ribosylamine-5P	K00764
2	*purD*	CTR2_1825	phosphoribosylamine---glycine ligase [EC:6.3.4.13]	5-phospho-D-ribosylamine	N1-(5-phospho-D-ribosyl)glycinamide	K01945
3	*purN*	CTR2_3886	phosphoribosylglycinamide formyltransferase 1 [EC:2.1.2.2]	GAR	N2-formyl-N1-(5-phospho-D-ribosyl)glycinamide	K11175
4	*purT*	CTR2_3758	phosphoribosylglycinamide formyltransferase 2 [EC:6.3.1.21]	FGAR	N1-(5-phospho-beta-D-ribosyl)glycinamide	K08289
5	*purL*	CTR2_3415	phosphoribosylformylglycinamidine synthase [EC:6.3.5.3]	N2-formyl-N1-(5-phospho-D-ribosyl)glycinamide	2-(formamido)-N1-(5-phospho-D-ribosyl)acetamidine	K01952
6	*purM*	CTR2_1075	phosphoribosylformylglycinamidine cyclo-ligase [EC:6.3.3.1]	2-(formamido)-N1-(5-phospho-D-ribosyl)acetamidine	5-amino-1-(5-phospho-D-ribosyl)imidazole	K01933
7	*purK*	CTR2_5038	5-(carboxyamino)imidazole ribonucleotide synthase [EC:6.3.4.18]	5-amino-1-(5-phospho-D-ribosyl)imidazole	5-Carbosyamino-1-(5-phospho-D-ribosyl)imidazole	K01589
8	*purE*	CTR2_5039	5-(carboxyamino)imidazole ribonucleotide mutase [EC:5.4.99.18]	5-Carbosyamino-1-(5-phospho-D-ribosyl)imidazole	5-amino-1-(5-phospho-D-ribosyle)imidazole-4-carboxylate	K01588
9	*purC*	CTR2_4964	phosphoribosylaminoimidazole-succinocarboxamide synthase [EC:6.3.2.6]	5-amino-1-(5-phospho-D-ribosyle)imidazole-4-carboxylate	(S)-2-[5-amino-1-(5-phospho-D-ribosyl)imidazole-4-carboxamido]succinate	K01923
10	*purB*	CTR2_4865	adenylosuccinate lyase [EC:4.3.2.2]	(S)-2-[5-amino-1-(5-phospho-D-ribosyl)imidazole-4-carboxamido]succinate	5-amino-1-(5-phopho-D-ribosyl)imidazole-4-carboxamide	K01756
11	*purH*	CTR2_0586	phosphoribosylaminoimidazolecarboxamide formyltransferase / IMP cyclohydrolase [EC:2.1.2.3 3.5.4.10]	5-amino-1-(5-phopho-D-ribosyl)imidazole-4-carboxamide	5-formamido-1-(5-phospho-D-ribosyl)imidazole-4-carboxamide	K00602

Transcripts per million values of target genes in Reactor I									
No.	I-1	I-2	I-3	I-4	I-5	log_2FC	logCPM	FDR	Fold[a]
1	498.902	448.429	326.529	315.305	176.471	-1.0510754	9.39611399	0.66195823	0.35371877
2	333.539	335.808	231.104	262.765	158.994	-0.6206335	8.74718353	0.99165211	0.47668788
3	82.688	94.237	68.739	64.016	32.607	-0.8940592	5.48194665	0.82846204	0.39433775
4	59.359	48.904	45.444	45.255	35.449	-0.2954274	6.32799834	0.99964082	0.59719672
5	172.497	176.51	131.961	157.598	82.472	-0.616358	9.43052895	0.99527205	0.47810687
6	251.777	230.732	159.266	204.136	116.784	-0.6600497	8.03037418	0.97854806	0.46383903
7	234.991	198.629	136.221	172.441	160.762	-0.0994452	8.32972236	0.99964082	0.68411982
8	263.346	225.438	182.934	251.655	220.976	0.19514861	7.39948687	0.99964082	0.839109
9	352.858	296.134	242.315	288.974	214.62	-0.2690604	8.48317445	0.99964082	0.60823334
10	127.608	126.539	86.699	103.634	56	-0.7399356	7.41044773	0.9295711	0.43884396
11	216.942	200.717	111.457	127.906	82.327	-0.9495999	8.31268188	0.76710425	0.37948853

[a]; Fold was calculated using the equation as follows:
Transcripts per million [TMP] values of I-5 / TMP value of I-1

Transcripts per million values of target genes in Reactor II									
No.	II-1	II-2	II-3	II-4	II-5	log_2FC	logCPM	FDR	Fold[a]
1	321.712	197.4	370.429	328.483	155.368	-0.735355	8.9241662	0.97644934	0.48294126
2	279.911	213.206	252.021	227.238	167.888	-0.4227569	8.62594114	0.99774764	0.59979065
3	72.577	51.93	74.935	121.2	50.157	-0.2182724	5.61814406	0.99774764	0.69108671
4	60.411	40.075	64.934	59.624	28.285	-0.7798615	6.21001791	0.96412124	0.46820943
5	125.917	159.731	133.357	118.273	98.886	-0.0339418	9.28513446	0.99796097	0.78532684
6	225.939	181.285	184.717	210.507	113.592	-0.6773258	7.91842507	0.99287377	0.50275517
7	153.129	190.011	151.009	189.499	117.007	-0.0734478	7.77547761	0.99774764	0.76410739
8	191.009	170.926	204.085	245.031	148.414	-0.0493101	6.86305631	0.99774764	0.77700004
9	287.473	219.401	255.939	316.425	123.363	-0.9057602	7.98444083	0.87108397	0.429129
10	136.57	122.958	108.441	132.465	66.881	-0.7152212	7.56852103	0.97756697	0.48971956
11	105.071	101.016	96.004	105.963	53.823	-0.6503163	7.4278732	0.99774764	0.51225362

[b]; Fold was calculated using the equation as follows:
Transcripts per million [TMP] values of II-5 / TMP value of II-1

Table S3-2 Adenine ribonucleotide synthesis

Adenine ribonucleotide synthesis						
No.	gene	locus-tag	Enzymes	Substrates	Products	ko number
1	*purA*	CTR2_1464	adenylosuccinate synthase [EC:6.3.4.4]	IMP	Adenylo-succinate	K01939
2	*purB*	CTR2_4865	adenylosuccinate lyase [EC:4.3.2.2]	Adenylo-succinate	AMP	K01756
3	*adk*	CTR2_1904	adenylate kinase [EC:2.7.4.3]	AMP	ADP	K00939
4	*ndk*	CTR2_1448	nucleoside-diphosphate kinase [EC:2.7.4.6]	ADP	ATP	K00940
5	*pyk*	CTR2_4991	pyruvate kinase [EC:2.7.1.40]			K00873

Transcripts per million values of target genes in Reactor I									
No.	II-1	II-2	II-3	II-4	II-5	log_2FC	logCPM	FDR	Fold[a]
1	398.944	384.958	297.769	333.474	323.344	0.14511738	9.44740911	0.99964082	0.81049972
2	127.61	126.54	86.699	103.63	56	-0.7399356	7.41044773	0.9295711	0.43883708
3	182.394	163.161	125.101	155.297	93.028	-0.523051	6.94067006	0.99964082	0.51003871
4	956.392	708.102	639.988	654.247	588.548	-0.252204	8.82155121	0.99964082	0.61538365
5	100.742	95.336	102.348	110.515	87.417	0.24353155	7.57660787	0.99964082	0.86773143

[a]; Fold was calculated using the equation as follows:

Transcripts per million [TMP] values of I-5 / TMP value of I-1

Transcripts per million values of target genes in Reactor II									
No.	II-1	II-2	II-3	II-4	II-5	log_2FC	logCPM	FDR	Fold[a]
1	306.374	315.767	328.102	263.979	156.298	-0.6562725	8.7519195	0.99774764	0.51015426
2	136.57	122.96	108.44	132.47	66.881	-0.7152212	7.56852103	0.97756697	0.48971956
3	202.701	152.504	187.062	217.579	123.929	-0.3951003	7.1975233	0.99774764	0.6113882
4	687.905	618.812	854.593	749.421	398.446	-0.4731018	8.30044638	0.99774764	0.57921661
5	104.768	138.964	126.976	146.159	76.686	-0.1354671	7.49139871	0.99774764	0.73196014

[b]; Fold was calculated using the equation as follows:

Transcripts per million [TMP] values of II-5 / TMP value of II-1

Table S3-3 Guanine ribonucleotide synthesis

Guanine ribonucleotide synthesis						
No.	gene	locus-tag	Products (Enzymes)	Substrate	Metabolite	ko number
1	*guaB*	CTR2_2384	IMP dehydrogenase [EC:1.1.1.205]	IMP	XMP	K00088
2	*guaA*	CTR2_2381	GMP synthase (glutamine-hydrolysing) [EC:6.3.5.2]	XMP	GMP	K01951
3	*gmk*	CTR2_0812	guanylate kinase [EC:2.7.4.8]	GMP	GDP	K00942
4	*ndk*	CTR2_1448	nucleoside-diphosphate kinase [EC:2.7.4.6]	GDP	GTP	K00940
5	*pyk*	CTR2_4991	pyruvate kinase [EC:2.7.1.40]			K00873

Transcripts per million values of target genes in Reactor I									
No.	II-1	II-2	II-3	II-4	II-5	log_2FC	logCPM	FDR	Fold[a]
1	752.75	599.6	519.22	609.99	417.62	-0.4017453	10.2022138	0.99964082	0.55479243
2	308.682	312.952	259.062	344.77	340.746	0.59080294	9.57338374	0.99964082	1.10387389
3	189.464	134.324	106.685	119.166	73.011	-0.9274153	6.83722416	0.78948627	0.38535553
4	956.392	708.102	639.988	654.247	588.548	-0.252204	8.82155121	0.99964082	0.61538365
5	100.742	95.336	102.348	110.515	87.417	0.24353155	7.57660787	0.99964082	0.86773143

[a]; Fold was calculated using the equation as follows:
Transcripts per million [TMP] values of I-5 / TMP value of I-1

Transcripts per million values of target genes in Reactor II									
No.	II-1	II-2	II-3	II-4	II-5	log_2FC	logCPM	FDR	Fold[a]
1	515.62	674.09	483.96	662.6	261.89	-0.6626259	9.60005958	0.9961345	0.5079128
2	324.182	303.347	236.676	450.318	275.292	0.07884965	9.4088848	0.99774764	0.84918965
3	123.072	95.513	144.77	136.445	69.751	-0.5044253	6.42752932	0.99774764	0.56674954
4	687.905	618.812	854.593	749.421	398.446	-0.4731018	8.30044638	0.99774764	0.57921661
5	104.768	138.964	126.976	146.159	76.686	-0.1354671	7.49139871	0.99774764	0.73196014

[b]; Fold was calculated using the equation as follows:
Transcripts per million [TMP] values of II-5 / TMP value of II-1

Table S3-4 Pyrimidine ribonucleotide biosynthesis

Pyrimidine ribonucleotide biosynthesis						
No.	gene	locus-tag	Products (Enzymes)	Substrate	Metabolite	ko number
1	*pyrH*	CTR2_1515	uridylate kinase [EC:2.7.4.22]	ATP, UMP	ADP, UDP	K09903
2	*ndk*	CTR2_1448	nucleoside-diphosphate kinase [EC:2.7.4.6]	ATP, nucleoside diphosphate	ADP, nucleoside triphosphate	K00940
3	*pyrG*	CTR2_3615	CTP synthase [EC:6.3.4.2]	ATP, UTP, L-glutamine	ADP, CTP, L-glutamate	K01937

Transcripts per million values of target genes in Reactor I									
No.	II-1	II-2	II-3	II-4	II-5	log_2FC	logCPM	FDR	Fold[a]
1	226.915	207.573	114.763	116.245	109.734	-0.5998734	7.36226263	0.99964082	0.48359077
2	956.392	956.392	639.988	654.247	588.548	-0.252204	8.82155121	0.99964082	0.61538365
3	453.459	453.459	313.547	383.063	293.003	-0.1818231	9.74117235	0.99964082	0.64615103

[a]; Fold was calculated using the equation as follows:
Transcripts per million [TMP] values of I-5 / TMP value of I-1

Transcripts per million values of target genes in Reactor II									
No.	II-1	II-2	II-3	II-4	II-5	log_2FC	logCPM	FDR	Fold[a]
1	129.025	170.7	113.409	104.079	125.638	0.27627903	7.0118359	0.99774764	0.97374927
2	687.905	618.812	854.593	749.421	398.446	-0.4731018	8.30044638	0.99774764	0.57921661
3	309.443	222.317	378.805	382.318	193.646	-0.3615463	9.15889812	0.99774764	0.62578892

[b]; Fold was calculated using the equation as follows:
Transcripts per million [TMP] values of II-5 / TMP value of II-1

Table S3-5 Pyrimidine deoxyribonucleotide biosynthesis

Pyrimidine deoxyribonucleotide biosynthesis (UDP to dTTP)						
No.	gene	locus-tag	Products (Enzymes)	Substrate	Metabolite	ko number
1	*nrdA, nrdE*	CTR2_0509	ribonucleoside-diphosphate reductase alpha chain [EC:1.17.4.1]	2'-deoxyribonucleoside 5'-dephosphate	ribonucleside 5'-diphasphate	K00525
2	*nrdB, nrdF*	CTR2_0507	ribonucleoside-diphosphate reductase beta chain [EC:1.17.4.1]	2'-deoxyribonucleoside 5'-dephosphate	ribonucleside 5'-diphasphate	K00526
3	*tmk*	CTR2_3509	dTMP kinase [EC:2.7.4.9]	ATP, dTMP	ADP, dTDP	K00943
4	*ndk*	CTR2_1448	nucleoside-diphosphate kinase [EC:2.7.4.6]	ATP, nucleoside diphosphate	ADP, nucleosde triphosphate	K00940
5	*dut*	CTR2_3611	dUTP pyrophosphatase [EC:3.6.1.23]	dUTP	dUMP	K01520
6	*thyA*	CTR2_1485	thymidylate synthase [EC:2.1.1.45]	5, 10-methylenetetrahydrofolate, dUMP	dihydrofolate, dTMP	K00560
7	*tmk*	CTR2_3509	dTMP kinase [EC:2.7.4.9]	ATP, dTMP	ADP, dTDP	K00943
8	*ndk*	CTR2_1448	nucleoside-diphosphate kinase [EC:2.7.4.6]	ATP, nucleoside diphosphate	ADP, nucleoside triphosphate	K00940

Transcripts per million values of target genes in Reactor I									
No.	II-1	II-2	II-3	II-4	II-5	log_2FC	logCPM	FDR	Fold[a]
1	263.091	167.168	327.911	316.231	102.82	-0.9071988	9.46870992	0.80894178	0.39081535
2	134.619	60.339	132.449	102.259	32.757	-1.5906295	6.94665709	0.18544954	0.24333118
3	62.173	57.871	49.036	55.388	33.217	-0.4560234	5.49071352	0.99964082	0.53426729
4	956.392	708.102	639.988	654.247	588.548	-0.252204	8.82155121	0.99964082	0.61538365
5	65.731	53.956	54.11	59.181	35.424	-0.443462	4.96254107	0.99964082	0.5389238
6	117.997	111.133	88.728	80.962	69.642	-0.3124522	6.83219079	0.99964082	0.59020145
7	62.173	57.871	49.036	55.388	33.217	-0.4560234	5.49071352	0.99964082	0.53426729
8	956.392	708.102	639.988	654.247	588.548	-0.252204	8.82155121	0.99964082	0.61538365

[a]; Fold was calculated using the equation as follows:

Transcripts per million [TMP] values of I-5 / TMP value of I-1

Transcripts per million values of target genes in Reactor II									
No.	II-1	II-2	II-3	II-4	II-5	log_2FC	logCPM	FDR	Fold[a]
1	201.624	416.11	206.935	353.476	79.793	-1.0226128	9.09627524	0.77805814	0.3957515
2	72.458	163.689	93.458	72.278	28.924	-1.009942	6.27531114	0.80015795	0.39918297
3	47.622	22.97	60.216	42.152	35.362	-0.1146812	5.31356005	0.99774764	0.74255596
4	687.905	618.812	854.593	749.421	398.446	-0.4731018	8.30044638	0.99774764	0.57921661
5	80.568	34.444	64.754	68.484	35.067	-0.8849346	5.14270891	0.91007236	0.43524724
6	119.241	64.13	87.767	126.842	53.792	-0.8335734	6.69096914	0.93389157	0.45112
7	47.622	22.97	60.216	42.152	35.362	-0.1146812	5.31356005	0.99774764	0.74255596
8	687.905	618.812	854.593	749.421	398.446	-0.4731018	8.30044638	0.99774764	0.57921661

[b]; Fold was calculated using the equation as follows:

Transcripts per million [TMP] values of II-5 / TMP value of II-1

Supplementary Table S4-1 Phenol and catechol degradation

Phenol and catechol degrading pathways						
No[a].	gene	locus-tag	Enzymes	Substrates	Products	ko number
1	*dmpK*	CTR2_1592	phenol/toluene 2-monooxygenase (NADH) P0/A0	Phenol	catechol	K16249
2	*dmpB*	CTR2_1599	catechol 2,3-dioxygenase [EC:1.13.11.2]	catechol	2-Hydroxymuconic semialdehyde	K00446
3	*dmpC*	CTR2_1603	aminomuconate-semialdehyde/2-hydroxymuconate-6-semialdehyde dehydrogenase [EC:1.2.1.85]	2-Hydroxymuconate semialdehyde	2-Hydroxymuconate	K10217
4	*praC*	CTR2_0002	4-oxalocrotonate tautomerase [EC:5.3.2.6]	2-Hydroxymuconate	gamma-oxalocrotonate	K01821
5	*dmpH*	CTR2_1611	2-oxo-3-hexenedioate decarboxylase [EC:4.1.1.77]	gamma-oxalocrotonate	2-Oxopent-4-enoate	K01617
6	*bphH*	CTR2_1604	2-oxopent-4-enoate/cis-2-oxohex-4-enoate hydratase [EC:4.2.1.80]	2-Oxopent-4-enoate	4-hydroxy-2-oxopentanoate	K18364
7	*bphI*	CTR2_1607	4-hydroxy-2-oxovalerate/4-hydroxy-2-oxohexanoate aldolase [EC:4.1.3.39]	4-hydroxy-2-oxopentanoate	acetaldehyde / pyruvate	K18365
8	*bphJ*	CTR2_1606	acetaldehyde/propanal dehydrogenase [EC:1.2.1.10]	acetaldehyde	Acetyl-CoA	K18366

[a]; The number corresponds to the number shown below the horizontal axis in figures 3A-I and 3A-II.

Transcripts per million values of target genes in Reactor I									
No.	I-1	I-2	I-3	I-4	I-5	log_2FC	logCPM	FDR	Fold[a]
1	1161	1849	8453	9696	35434	5.38	12.49	8.8E-11	31
2	2908	4285	7084	6865	9656	2.18	13.10	0.02037	3.3
3	1993	1293	2390	2174	1639	0.17	11.92	0.99964	0.82
4	68	77	59	46	57	0.19	4.07	0.99964	0.83
5	2118	1253	2064	1709	449	-1.79	10.35	0.0985	0.21
6	1736	1073	1644	1515	991	-0.36	10.51	0.99964	0.57
7	1588	903	1440	1124	466	-1.32	10.44	0.38811	0.29
8	1742	776	1282	1140	606	-1.07	10.48	0.63674	0.35

[a]; Fold was calculated using the equation as follows:
Transcripts per million [TMP] values of I-5 / TMP value of I-1

Transcripts per million values of target genes in Reactor II									
No.	II-1	II-2	II-3	II-4	II-5	log_2FC	logCPM	FDR	Fold[b]
1	1679	21811	2501	2034	25714	4.25	11.98	1.1425E-06	15
2	7590	15956	12774	4992	8979	0.56	13.37	0.99774764	1.2
3	4363	3465	4816	1772	1330	-1.40	12.48	0.42808553	0.30
4	75	47	96	111	60	0.00	4.17	0.99907837	0.81
5	4770	1135	4164	1986	519	-2.88	11.39	0.00227906	0.11
6	2879	1863	3322	1239	639	-1.86	10.81	0.13755177	0.22
7	3810	933	2724	1118	390	-2.97	11.44	0.00145737	0.10
8	2926	1446	3117	1398	459	-2.36	10.98	0.02240039	0.16

[b]; Fold was calculated using the equation as follows:
Transcripts per million [TMP] values of II-5 / TMP value of II-1

Supplementary Table S4-2 TCA cycle

TCA cycle						
No[a].	gene	locus-tag	Enzymes	Substrates	Products	ko number
1	*gltA*	CTR2_1354	citrate synthase [EC:2.3.3.1]	Acetyl-CoA & Oxaloacetate	Citrate	K01647
2	*acnA*	CTR2_1384	aconitate hydratase [EC:4.2.1.3]	Citrate	isocitrate	K01681
3	*acnB*	CTR2_1366	aconitate hydratase 2 / 2-methylisocitrate dehydratase [EC:4.2.1.3 4.2.1.99]			K01682
4	*icd*	CTR2_1985	isocitrate dehydrogenase [EC:1.1.1.42]	isocitrate	2-oxoglutarate	K00031
5	*sucA*	CTR2_2972	2-oxoglutarate dehydrogenase E1 component [EC:1.2.4.2]	2-oxoglutarate	s-sccinyldihydrolipoyllysine	K00164
6	*sucB*	CTR2_2971	2-oxoglutarate dehydrogenase E2 component (dihydrolipoamide succinyltransferase) [EC:2.3.1.61]	s-sccinyldihydrolipoyllysine	succinyl-CoA	K00658
7	*sucD*	CTR2_4842	succinyl-CoA synthetase alpha subunit [EC:6.2.1.5]	succinyl-CoA	Succinate and CoA	K01902
8	*sucC*	CTR2_4841	succinyl-CoA synthetase beta subunit [EC:6.2.1.5]		Succinate	K01903
9	*sdhA/frdA*	CTR2_1357	succinate dehydrogenase / fumarate reductase, flavoprotein subunit [EC:1.3.5.1 1.3.5.4]	Succinate	Fumarate	K00239
10	*sdhB/frdB*	CTR2_1356	succinate dehydrogenase / fumarate reductase, iron-sulfur subunit [EC:1.3.5.1 1.3.5.4]			K00240
11	*sdhC/frdC*	CTR2_1359	succinate dehydrogenase / fumarate reductase, cytochrome b subunit			K00241
12	*sdhD/frdD*	CTR2_1358	succinate dehydrogenase / fumarate reductase, membrane anchor subunit			K00242
13	*fumA/fumB*	CTR2_5301	fumarate hydratase, class I [EC:4.2.1.2]	Fumarate	Malate	K01676
14	*fumC*	CTR2_5299	fumarate hydratase, class II [EC:4.2.1.2]			K01679
15	*mdh*	CTR2_1362	malate dehydrogenase [EC:1.1.1.37]	Malate	Oxaloacetate	K00024
16	*mqo*	CTR2_1070	malate dehydrogenase (quinone) [EC:1.1.5.4]			K00116

[a]; The number corresponds to the number shown below the horizontal axis in figures 3B-I and 3B-II.

Transcripts per million values of target genes in Reactor I									
No[a].	I-1	I-2	I-3	I-4	I-5	log_2FC	logCPM	FDR	Fold[b]
1	870.019	635.208	679.318	633.426	733.262	0.20151402	10.5311426	0.99964082	0.84
2	94.724	77.903	83.95	86.9	110.719	0.67332525	8.75962228	0.96967484	1.2
3	216.537	166.681	219.939	188.38	151.807	-0.0641348	9.36900642	0.99964082	0.70
4	141.306	113.562	118.887	114.923	102.368	-0.0168259	7.74337938	0.99964082	0.72
5	672.109	528.744	528.831	530.927	218.724	-1.1713424	10.7201209	0.52258584	0.33
6	618.444	384.337	478.117	380.953	187.141	-1.2762593	9.35562652	0.43820079	0.30
7	563.14	578.385	788.014	544.609	217.199	-0.926223	8.86731407	0.78919246	0.39
8	555.354	476.43	566.686	460.458	219.686	-0.8897169	9.22710208	0.82362847	0.40
9	342.761	229.336	285.596	290.295	259.878	0.04886473	9.56995322	0.99964082	0.76
10	357.568	311.783	328.251	315.367	263.312	0.00678941	8.25307124	0.99964082	0.74
11	411.599	294.413	323.552	342.617	260.734	-0.210419	7.62513936	0.99964082	0.63
12	245.301	145.272	179.279	167.892	182.247	0.01957387	6.77133612	0.99964082	0.74
13	178.323	109.301	98.67	135.838	190.837	0.5460683	8.68454065	0.99964082	1.07
14	940.521	672.828	543.177	428.935	207.769	-1.7302242	10.0062033	0.11748345	0.22
15	1038.255	625.828	905.113	1054.189	936.364	0.2992123	10.4340903	0.99964082	0.90
16	19.185	18.625	18.721	19.895	21.54	0.61516111	5.63688047	0.99805165	1.1

[b]; Fold was calculated using the equation as follows:
Transcripts per million [TMP] values of I-5 / TMP value of I-1

Transcripts per million values of target genes in Reactor II									
No[a].	II-1	II-2	II-3	II-4	II-5	log_2FC	logCPM	FDR	Fold[c]
1	1114.937	688.218	780.46	846.534	573.926	-0.6433216	10.5487725	0.99774764	0.51
2	168.844	130.878	87.329	132.449	125.165	-0.1171587	9.21089955	0.99774764	0.74
3	402.289	144.774	274.379	194.407	128.916	-1.3270775	9.82863915	0.48949434	0.32
4	151.973	116.079	120.452	166.589	128.723	0.07514566	7.94515634	0.99774764	0.85
5	705.685	354.153	458.524	634.771	300.578	-0.916582	10.9236627	0.86530116	0.43
6	633.434	256.632	400.031	431.585	236.495	-1.1066627	9.49200419	0.69795403	0.37
7	1026.851	146.387	972.228	387.661	226.318	-1.8670551	9.52372819	0.13608461	0.22
8	740.492	140.681	817.638	497.301	237.118	-1.3281486	9.55339862	0.48949434	0.32
9	445.415	218.916	267.113	256.988	195.167	-0.8757232	9.60126388	0.90035832	0.44
10	555.833	234.403	372.121	270.178	238.506	-0.9058852	8.55395336	0.8695151	0.43
11	443.337	296.275	327.553	406.16	352.376	-0.0165909	7.87565666	0.99907837	0.79
12	243.008	166.691	198.662	192.073	147.101	-0.409436	6.61065628	0.99774764	0.61
13	121.177	178.972	108.662	154.428	133.632	0.45582035	8.12483235	0.99774764	1.1
14	649.442	150.112	494.477	329.419	249.021	-1.068215	9.70466989	0.74147772	0.38
15	1080.148	657.216	874.28	819.228	911.646	0.07001167	10.419296	0.99774764	0.84
16	21.11	10.382	20.771	24.972	21.515	0.34195505	5.66919524	0.99774764	1.0

[c]; Fold was calculated using the equation as follows:
Transcripts per million [TMP] values of II-5 / TMP value of II-1

Supplementary Table S4-3 Glyoxylate shunt

Glyoxylate shunt

No[a].	gene	locus-tag	Enzymes	Substrates	Products	ko number
1	*gltA*	CTR2_1354	citrate synthase [EC:2.3.3.1]	Oxaloacetate	Citrate	K01647
2	*acnA*	CTR2_1384	aconitate hydratase [EC:4.2.1.3]	Citrate	Isocitrate	K01681
3	*acnB*	CTR2_1366	aconitate hydratase 2 / 2-methylisocitrate dehydratase [EC:4.2.1.3][EC: 4.2.1.99]			K01682
4	*aceA*	CTR2_1688	isocitrate lyase [EC:4.1.3.1]	Isocitrate	Glyoxylate	K01637
5	*aceB*	CTR2_0005	malate synthase [EC:2.3.3.9]	Glyoxylate	Malate	K01638
6	*mdh*	CTR2_1362	malate dehydrogenase [EC:1.1.1.37]	Malate	Oxaloacetate	K00024

[a]; The number corresponds to the number shown below the horizontal axis in figures 3C-I and 3C-II.

Transcripts per million values of target genes in Reactor I

No[a].	I-1	I-2	I-3	I-4	I-5	log_2FC	logCPM	FDR	Fold[b]
1	870.019	635.208	679.318	633.426	733.262	0.20151402	10.5311426	0.99964082	0.84
2	94.724	77.903	83.95	86.9	110.719	0.67332525	8.75962228	0.96967484	1.2
3	216.537	166.681	219.939	188.38	151.807	-0.0641348	9.36900642	0.99964082	0.70
4	5861.127	2023.639	2740.762	1213.686	4860.042	0.1780238	13.2935037	0.99964082	0.83
5	161.449	162.557	261.507	227.997	160.574	0.44037868	8.97426102	0.99964082	0.99
6	1038.255	625.828	905.113	1054.189	936.364	0.2992123	10.4340903	0.99964082	0.90

[b]; Fold was calculated using the equation as follows:

Transcripts per million [TMP] values of I-5 / TMP value of I-1

Transcripts per million values of target genes in Reactor II

No[a].	II-1	II-2	II-3	II-4	II-5	log_2FC	logCPM	FDR	Fold[c]
1	1114.9	688.22	780.46	846.53	573.93	-0.6433216	10.5487725	0.99774764	0.51
2	168.844	130.878	87.329	132.449	125.165	-0.1171587	9.21089955	0.99774764	0.74
3	402.289	144.774	274.379	194.407	128.916	-1.3270775	9.82863915	0.48949434	0.32
4	4028.473	564.284	3572.844	2892.415	8274.458	1.35312485	13.5404076	0.46680246	2.1
5	207.606	188.646	166.635	257.817	161.491	-0.0476993	9.12678072	0.99774764	0.78
6	1080.148	657.216	874.28	819.228	911.646	0.07001167	10.419296	0.99774764	0.84

[c]; Fold was calculated using the equation as follows:

Transcripts per million [TMP] values of II-5 / TMP value of II-1

Supplementary Table S4-4 GS-GOGAT and GDH

GS-GOGAT and GDH						
No[a].	gene	locus-tag	Enzymes	Substrates	Products	ko number
1	*glnA*	CTR2_1472	glutamine synthetase[b] [EC:6.3.1.2]	L-glutamate, NH_3, ATP	L-glutamine	K01915
2	*gltD*	CTR2_4073	glutamate synthase[c] (NADPH) small chain [EC:1.4.1.13]	L-glutamine, 2-OG, NADPH	L-glutamate[e]	K00266
3	*gltB*	CTR2_4074	glutamate synthase[c] (NADPH) large chain [EC:1.4.1.13]	L-glutamine, 2-OG, NADPH	L-glutamate[e]	K00265
4	*gdhA*	CTR2_3645	glutamate dehydrogenase[d] (NADP+) [EC:1.4.1.4]	2-OG, NH_3, NADPH	L-glutamate, NADP+	K00262
5	*gdhA*	CTR2_4700	glutamate dehydrogenase[d] (NAD+) [EC:1.4.1.3]	L-glutamate, NAD+	2-OG, NH_3, NADH	K00261

[a]; The number corresponds to the number shown below the horizontal axis in figures 3E-I and 3E-II.

[b]; Glutamine synthetase (GS), [c]; Glutamate synthase (GOGAT), [d]; Glutamate dehydrogenase (GDH)

[e]; 2 mol of L-glutamate are produced from L-glutamine and 2-OG.

Transcripts per million values of target genes in Reactor I									
No[a].	I-1	I-2	I-3	I-4	I-5	log_2FC	logCPM	FDR	Fold[f]
1	260.813	204.438	336.977	526.532	531.5	1.47526718	9.7183753	0.2610355	2.0
2	134.05	183.927	166.575	201.95	270.202	1.45946629	8.80638814	0.27132612	2.0
3	203.949	209.732	264.671	391.888	459.684	1.62065758	11.2140529	0.16504748	2.3
4	304.086	158.368	33.685	61.667	104.383	-1.0943275	8.50458353	0.62106968	0.34
5	568.62	1494.342	1826.262	1341.015	1578.842	1.92155127	11.1372097	0.05877007	0.51

[f]; Fold was calculated using the equation as follows:

Transcripts per million [TMP] values of I-5 / TMP value of I-1

Transcripts per million values of target genes in Reactor II									
No[a].	II-1	II-2	II-3	II-4	II-5	log_2FC	logCPM	FDR	Fold[g]
1	278.97	1099.107	237.4	297.002	725.598	1.69372175	10.029196	0.20443219	2.6
2	257.034	1050.085	266.113	163.344	175.014	-0.2397849	8.77469868	0.99774764	4.1
3	228.895	1760.563	234.802	400.612	387.27	1.07334076	11.0379237	0.73725254	7.7
4	64.27	32.488	54.662	45.941	28.55	-0.8557947	6.3987067	0.92054075	0.51
5	1396.673	1242.118	963.94	1213.372	678.129	-0.7276619	10.9059595	0.97644934	0.47

[g]; Fold was calculated using the equation as follows:

Transcripts per million [TMP] values of II-5 / TMP value of II-1

Supplementary Table S4-5 Urea cycle

Urea cycle						
No[a].	gene	locus-tag	Enzymes	Substrates	Products	ko number
1	*argH*	CTR2_1809	argininosuccinate lyase [EC:4.3.2.1]	L-argininosuccinate	L-arginine and fumarate	K01755
2	*rocF*	CTR2_0414	arginase [EC:3.5.3.1]	L-arginine	L-ornithine and Urea	K01476
3	*argF*	CTR2_1066	ornithine carbamoyltransferase [EC:2.1.3.3]	L-ornithine	L-citrulline	K00611
4	*argG*	CTR2_5281	argininosuccinate synthase [EC:6.3.4.5]	L-citrulline and L-aspartate	L-argininosuccinate	K01940

[a]; The number corresponds to the number shown below the horizontal axis in figures 3D-I and 3D-II.

Transcripts per million values of target genes in Reactor I									
No[a].	I-1	I-2	I-3	I-4	I-5	log_2FC	logCPM	FDR	Fold[b]
1	22.744	20.675	16.329	18.014	16.835	0.01421471	5.45431893	0.99964082	0.7
2	93.413	69.667	52.174	50.393	77.927	0.18670855	6.8468286	0.99964082	0.8
3	219.415	174.137	166.315	172.496	115.756	-0.4743188	7.71836156	0.99964082	0.5
4	493.721	280.931	288.122	310.796	220.657	-0.7136491	9.32620459	0.95156613	0.4

[b]; Fold was calculated using the equation as follows:
Transcripts per million [TMP] values of I-5 / TMP value of I-1

Transcripts per million values of target genes in Reactor II									
No[a].	II-1	II-2	II-3	II-4	II-5	log_2FC	logCPM	FDR	Fold[c]
1	21.094	13.427	19.847	30.629	23.441	0.4667579	5.64092315	0.99774764	1.1
2	120.221	56.743	68.315	103.008	71.447	-0.4359938	6.96510359	0.99774764	0.6
3	251.413	197.135	223.139	199.445	127.441	-0.6654785	7.88861548	0.99546417	0.5
4	283.751	279.766	241.69	320.699	118.336	-0.9469887	8.49423524	0.84175536	0.4

[c]; Fold was calculated using the equation as follows:
Transcripts per million [TMP] values of II-5 / TMP value of II-1

Supplementary Table S5-1. Top 20 of up-regulated genes in reactor I

locus tag	Predicted product in reactor I	log_2FC[a]	FDR[b]	Transcripts per million in reactor I					ko number[c]
				I-1	I-2	I-3	I-4	I-5	
CTR2_4687	hypothetical protein CTR2_4687	8.20	4.0×10^{-19}	14	109	1035	1867	3084	N/A[d]
CTR2_4688	ammonium transporter, Amt family	8.04	4.0×10^{-19}	19	74	757	1580	3681	K03320
CTR2_4183	nitrate/nitrite transport system substrate-binding protein	7.98	4.0×10^{-19}	34	126	1588	2973	6247	K15576
CTR2_4182	nitrate/nitrite transport system permease protein	7.97	4.0×10^{-19}	20	105	1494	2471	3719	K15577
CTR2_4179	nitrite reductase (NADH) small subunit [EC:1.7.1.15]	7.85	1.5×10^{-18}	9	109	651	1033	1535	K00363
CTR2_4180	nitrite reductase (NADH) large subunit [EC:1.7.1.15]	7.72	1.8×10^{-18}	13	56	859	1377	1941	K00362
CTR2_4181	nitrate/nitrite transport system ATP-binding protein [EC:3.6.3.-]	7.65	3.3×10^{-18}	14	112	768	1255	2016	K15578
CTR2_4184	response regulator NasT	7.00	5.0×10^{-16}	8	27	172	369	750	N/A[d]
CTR2_0056	hypothetical protein CTR2_0056	6.02	8.1×10^{-9}	1	4	23	32	35	N/A[d]
CTR2_4686	glycerophosphoryl diester phosphodiesterase [EC:3.1.4.46]	5.43	7.5×10^{-11}	26	43	270	504	807	K01126
CTR2_1592	phenol/toluene 2-monooxygenase (NADH) P0/A0	5.38	8.8×10^{-11}	1161	1849	8453	9696	35434	K16249
CTR2_4612	probable signal peptide protein	5.36	1.2×10^{-10}	12	25	261	380	371	N/A[d]
CTR2_4178	assimilatory nitrate reductase catalytic subunit [EC:1.7.99.-]	5.34	1.1×10^{-10}	16	73	206	353	464	K00372
CTR2_4177	uroporphyrin-III C-methyltransferase [EC:2.1.1.107]	5.27	1.8×10^{-10}	21	102	300	501	607	K02303
CTR2_0048	urease subunit gamma [EC:3.5.1.5]	5.27	3.3×10^{-10}	11	21	171	238	315	K01430
CTR2_4856	type IV pilus assembly protein PilA	5.16	3.3×10^{-10}	170	258	2426	3188	4437	K02650
CTR2_5270	outer membrane protein, heavy metal efflux system	4.98	1.3×10^{-9}	14	21	82	175	332	K15725
CTR2_0054	urea transport system substrate-binding protein	4.98	1.7×10^{-9}	6	9	72	128	146	K11959
CTR2_1593	phenol/toluene 2-monooxygenase (NADH) P1/A1 [EC:1.14.13.244 1.14.13.243]	4.85	2.7×10^{-9}	1396	3325	12543	13758	40953	K16243
CTR2_0055	urea transport system permease protein	4.85	5.5×10^{-9}	2	4	24	33	47	K11960

[a]; log_2FC: log_2 fold-change from I-1 to I-5., [b]; FDR: false discovery rate, [c]; ko number: annotation number on KEGG, [d]; N/A: Not annotated.

Supplementary Table S5-2. Top 20 of up-regulated genes in reactor II

locus tag	Predicted product in reactor II	log_2FC[a]	FDR[b]	Transcripts per million in reactor II					ko number[c]
				II-1	II-2	II-3	II-4	II-5	
CTR2_4183	nitrate/nitrite transport system substrate-binding protein	7.38	1.4×10^{-14}	62	5620	173	210	8297	K15576
CTR2_4180	nitrite reductase (NADH) large subunit [EC:1.7.1.15]	7.25	1.7×10^{-14}	24	2790	71	103	2889	K00362
CTR2_4688	ammonium transporter, Amt family	7.11	3.4×10^{-14}	26	3216	83	111	2833	K03320
CTR2_4687	hypothetical protein CTR2_4687	7.07	4.0×10^{-14}	36	4358	107	155	3920	N/A[d]
CTR2_4184	response regulator NasT	6.80	1.8×10^{-13}	9	836	21	27	809	N/A[d]
CTR2_4182	nitrate/nitrite transport system permease protein	6.79	1.8×10^{-13}	81	5411	283	206	7147	K15577
CTR2_4179	nitrite reductase (NADH) small subunit [EC:1.7.1.15]	6.65	4.8×10^{-13}	25	1478	137	162	2000	K00363
CTR2_0241	NitT/TauT family transport system substrate-binding protein	6.39	8.0×10^{-12}	2	47	5	8	156	K02051
CTR2_0056	hypothetical protein CTR2_0056	6.37	3.2×10^{-8}	1	74	3	8	156	N/A[d]
CTR2_4181	nitrate/nitrite transport system ATP-binding protein [EC:3.6.3.-]	6.09	1.4×10^{-11}	71	2425	257	183	3865	K15578
CTR2_4177	uroporphyrin-III C-methyltransferase [EC:2.1.1.107]	5.77	1.3×10^{-10}	22	257	124	161	7897	K02303
CTR2_4686	glycerophosphoryl diester phosphodiesterase [EC:3.1.4.46]	5.66	2.2×10^{-10}	30	1251	81	84	1223	K01126
CTR2_4856	type IV pilus assembly protein PilA	5.52	5.1×10^{-10}	214	9226	787	261	7897	K02650
CTR2_4612	probable signal peptide protein	5.49	7.9×10^{-10}	16	1551	32	42	579	N/A[d]
CTR2_0055	urea transport system permease protein	5.41	3.6×10^{-9}	2	101	4	5	55	K11960
CTR2_4178	assimilatory nitrate reductase catalytic subunit [EC:1.7.99.-]	5.20	3.8×10^{-9}	26	387	121	103	773	K00372
CTR2_0046	urease subunit beta [EC:3.5.1.5]	5.19	1.4×10^{-8}	8	324	16	49	223	K01429
CTR2_0240	cytidine/deoxycytidylate deaminase family protein	5.10	2.8×10^{-8}	4	38	9	7	109	N/A[d]
CTR2_0239	NitT/TauT family transport system substrate-binding protein	5.07	1.4×10^{-8}	7	76	8	10	177	K02051
CTR2_0048	urease subunit gamma [EC:3.5.1.5]	4.99	2.5×10^{-8}	15	414	18	39	374	K01430

[a]; log_2FC: log_2 fold-change from II-1 to II-5., [b]; FDR: false discovery rate, [c]; ko number: annotation number on KEGG, [d]; N/A: Not annotated.

Supplementary Table S5-3. Top 20 of down-regulated genes in reactor I

locus tag	Predicted product in reactor I	log_2FC[a]	FDR[b]	Transcripts per million in reactor I					ko number[c]
				I-1	I-2	I-3	I-4	I-5	
CTR2_1620	hypothetical protein CTR2_1620	-7.61	3.0×10^{-3}	13	7	11	11	0	N/A[d]
CTR2_3566	putative hemin transport protein	-3.80	2.9×10^{-6}	850	683	654	578	45	K07225
CTR2_3565	iron complex transport system substrate-binding protein	-3.61	9.4×10^{-6}	901	924	711	582	54	K02016
CTR2_3567	hypothetical protein CTR2_3567	-3.60	1.2×10^{-5}	648	537	577	498	39	N/A[d]
CTR2_3564	iron complex transport system permease protein	-3.45	2.5×10^{-5}	405	379	306	282	27	K02015
CTR2_5017	outer membrane porin protein 32 precursor	-3.11	2.3×10^{-4}	76	24	16	13	6	N/A[d]
CTR2_0328	catecholate siderophore receptor	-3.08	1.9×10^{-4}	1903	626	377	360	164	K16090
CTR2_1576	oxidoreductase probably involved in sulfite reduction	-3.04	3.5×10^{-4}	186	68	244	137	17	N/A[d]
CTR2_0096	nicotinamide-nucleotide amidase [EC:3.5.1.42]	-3.01	3.0×10^{-4}	704	271	166	186	64	K03743
CTR2_3563	iron complex transport system ATP-binding protein [EC:7.2.2.-]	-3.00	3.4×10^{-4}	273	247	179	182	25	K02013
CTR2_1574	sulfate adenylyltransferase subunit 2 [EC:2.7.7.4]	-2.93	5.6×10^{-4}	120	27	166	84	12	K00957
CTR2_4444	DNA-directed RNA polymerase subunit beta' [EC:2.7.7.6]	-2.88	5.8×10^{-4}	450	395	315	238	45	K03046
CTR2_0094	putative exported protein	-2.86	7.9×10^{-4}	300	126	79	86	30	N/A[d]
CTR2_0327	PKHD-type hydroxylase [EC:1.14.11.-]	-2.84	8.1×10^{-4}	299	119	57	52	31	K07336
CTR2_1575	phosphoadenosine phosphosulfate reductase [EC:1.8.4.8 1.8.4.10]	-2.77	1.4×10^{-3}	133	32	147	72	14	K00390
CTR2_3673	NADH-quinone oxidoreductase subunit N [EC:7.1.1.2]	-2.74	1.3×10^{-3}	170	94	94	78	19	K00343
CTR2_0093	putative lipoprotein	-2.74	1.3×10^{-3}	366	163	86	88	40	N/A[d]
CTR2_3196	pE_PGRS family protein	-2.67	1.7×10^{-3}	2823	882	1881	1888	325	N/A[d]
CTR2_3664	branched-chain amino acid transport system permease protein	-2.67	$2.\times10^{-3}$	194	78	73	40	22	K01997
CTR2_2703	hypothetical protein CTR2_2703	-2.64	4.0×10^{-2}	28	28	9	7	3	N/A[d]

[a]; log_2FC: log_2 fold-change from I-1 to I-5., [b]; FDR: false discovery rate, [c]; ko number: annotation number on KEGG, [d]; N/A: Not annotated.

Supplementary Table S5-4. Top 20 of down-regulated genes in reactor II

locus tag	Predicted product in reactor II	log_2FC[a]	FDR[b]	Transcripts per million in reactor II					ko number[c]
				II-1	II-2	II-3	II-4	II-5	
CTR2_4590	citrate lyase subunit beta / citryl-CoA lyase [EC:4.1.3.34]	-4.95	1.5×10^{-6}	36	2	34	14	1	K01644
CTR2_4591	putative tricarboxylic transport membrane protein	-4.10	1.7×10^{-5}	48	4	61	15	2	K07795
CTR2_1612	putative tricarboxylic transport membrane protein	-3.78	1.7×10^{-5}	5136	763	3347	1501	301	K07795
CTR2_1613	4-oxalocrotonate tautomerase [EC:5.3.2.6]	-3.74	2.1×10^{-5}	9024	1054	5544	2829	542	K01821
CTR2_4592	immune-responsive protein 1	-3.49	1.7×10^{-4}	60	5	39	11	4	N/A[d]
CTR2_1036	L-aspartate oxidase [EC:1.4.3.16]	-3.12	7.2×10^{-4}	703	237	315	294	65	K00278
CTR2_3665	branched-chain amino acid transport system substrate-binding protein	-3.11	7.7×10^{-4}	508	55	400	251	47	K01999
CTR2_3664	branched-chain amino acid transport system permease protein	-3.08	9.7×10^{-4}	318	30	284	179	30	K01997
CTR2_1607	4-hydroxy-2-oxovalerate/4-hydroxy-2-oxohexanoate aldolase [EC:4.1.3.39 4.1.3.43]	-2.97	1.5×10^{-3}	3810	933	2724	1118	390	K18365
CTR2_5193	alpha-ketoglutarate-dependent taurine dioxygenase	-2.90	2.9×10^{-3}	155	29	22	21	17	N/A[d]
CTR2_1611	2-oxo-3-hexenedioate decarboxylase [EC:4.1.1.77]	-2.88	2.3×10^{-3}	4770	1135	4164	1986	519	K01617
CTR2_0482	cell division protein FtsZ	-2.87	2.4×10^{-3}	1949	400	1105	1342	214	K03531
CTR2_0294	putative Mg2+ transporter-C (MgtC) family protein	-2.85	3.0×10^{-3}	383	61	183	283	43	K07507
CTR2_3909	putative tricarboxylic transport membrane protein	-2.84	2.9×10^{-3}	684	62	1187	255	77	K07793
CTR2_3982	glycerophosphoryl diester phosphodiesterase [EC:3.1.4.46]	-2.82	1.0×10^{-2}	26	6	14	6	3	K01126
CTR2_1610	hypothetical protein CTR2_1610	-2.80	3.6×10^{-3}	5693	1236	4825	2001	659	N/A[d]
CTR2_0295	P-type Mg2+ transporter [EC:7.2.2.14]	-2.72	5.0×10^{-3}	279	65	147	278	34	K01531
CTR2_3440	hypothetical protein CTR2_3440	-2.71	3.2×10^{-2}	63	7	32	11	8	N/A[d]
CTR2_0094	putative exported protein	-2.71	6.0×10^{-3}	427	32	322	11	8	N/A[d]
CTR2_5017	outer membrane porin protein 32 precursor	-2.70	7.0×10^{-3}	42	5	35	20	5	N/A[d]

[a]; log_2FC: log_2 fold-change from I-1 to I-5., [b]; FDR: false discovery rate, [c]; ko number: annotation number on KEGG, [d]; N/A: Not annotated.
